# CagriSema versus Semaglutide 2.4 mg monotherapy for weight management in adults with overweight or obesity, with or without type 2 diabetes: a systematic review, meta-analysis, and trial sequential analysis

**DOI:** 10.3389/fendo.2026.1903335

**Published:** 2026-10-02

**Authors:** Zehra Khatoon Rasool, Rajvi Shah, Ansharah E Zinnia Batool, Rutvi Shah, Mariam Khachatryan, Sushant Bhardwaj, Ahmad Ali, Meeran Atta, Pugazhandhi Bakthavatchalam, Mirza Muhammad Hadeed Khawar, Muneeb Khawar

**Affiliations:** 1 DePaul University, Chicago, IL, United States; 2 Narendra Modi Medical College, Ahmedabad, India; 3 Rashid Latif Medical College, Lahore, Pakistan; 4 Fanarjyan Clinic, Yerevan, Armenia; 5 Kathmandu University School of Medical Sciences, Dhulikhel, Nepal; 6 Services Institute of Medical Sciences, Lahore, Pakistan; 7 American University of Antigua, St. John's, Antigua and Barbuda; 8 King Edward Medical University, Lahore, Pakistan

**Keywords:** cagrilintide, CagriSema, obesity, Semaglutide, weight loss

## Abstract

**Introduction:**

Semaglutide 2.4 mg is the reference pharmacotherapy for weight management, yet many patients do not reach ambitious weight-loss targets. CagriSema, a fixed-dose combination of cagrilintide and semaglutide, may offer additional benefit through complementary mechanisms. We quantified its incremental efficacy and safety against semaglutide 2.4 mg monotherapy in adults with overweight or obesity, with and without type 2 diabetes.

**Methods:**

This PRISMA 2020–compliant review (PROSPERO CRD420261380458) searched PubMed, Embase, Cochrane CENTRAL, ScienceDirect, ClinicalTrials.gov and WHO ICTRP to 30 June 2026 for randomised controlled trials comparing CagriSema with semaglutide 2.4 mg or placebo. Effects were pooled within two prespecified comparator strata using random-effects models with restricted maximum likelihood estimation of τ² and a Hartung–Knapp–Sidik–Jonkman small-sample adjustment, with prediction intervals, leave-one-out and phase-3a-restricted sensitivity analyses, trial sequential analysis.

**Results:**

Five trials (4,746 unique participants) were included. Versus semaglutide 2.4 mg, CagriSema produced greater weight loss (mean difference −6.89 percentage points, 95% CI −8.70 to −5.06; I² = 33%; prediction interval −8.91 to −4.86), a greater absolute reduction in body weight (−7.16 kg, 95% CI −9.15 to −5.17) and in body-mass index (−2.41 kg/m², 95% CI −2.83 to −1.99), and roughly twice the likelihood of reaching a 20% reduction (RR 2.10, 95% CI 1.78 to 2.47). Waist circumference fell further (−4.07 cm, 95% CI −5.10 to −3.04). No incremental glycaemic benefit was detected, with intervals for glycated haemoglobin (−0.07, 95% CI −0.14 to 0.01) and fasting plasma glucose lying entirely within prespecified minimal important differences. Injection-site reactions were more frequent (RR 3.14, 95% CI 1.42 to 6.96); gastrointestinal adverse events were not significantly increased under the primary model (RR 1.09, 95% CI 0.97 to 1.21).

**Conclusion:**

CagriSema produces clinically meaningful additional weight loss and modest improvements in surrogate cardiometabolic measures compared with semaglutide 2.4 mg alone, at the cost of more frequent injection-site reactions and, at most, modestly more frequent gastrointestinal events. The evidence is insufficient to determine whether serious adverse events or neoplasms differ between treatments, and long-term durability, cardiovascular outcomes and body composition remain undetermined.

**Systematic review registration:**

https://www.crd.york.ac.uk/prospero/, identifier CRD420261380458.

## Introduction

Obesity is one of the most pressing chronic diseases of the modern era, and its prevalence continues to rise at an alarming pace. The World Obesity Atlas 2025 projects that the number of adults living with obesity will increase by more than 115% between 2010 and 2030, reaching an estimated 1.13 billion people worldwide (1). Global forecasting analyses further suggest that, on current trajectories, the combined burden of overweight and obesity could affect roughly half of the adult population within the coming decades (2). This escalating burden is tightly linked to type 2 diabetes, with overweight or obesity present in the large majority of affected individuals, positioning weight management as a central, and increasingly primary, therapeutic target in this population (3).

Sustained weight loss confers meaningful clinical benefit, including improved glycaemic control, favourable changes in cardiovascular risk factors, and the potential for disease modification in type 2 diabetes (3). Among available pharmacotherapies, the glucagon-like peptide-1 (GLP-1) receptor agonist semaglutide has transformed the treatment landscape. In adults with overweight or obesity, once-weekly subcutaneous semaglutide 2.4 mg produced clinically important weight reductions in the STEP programme (4), and in adults with type 2 diabetes it achieved a mean body weight reduction of approximately 9.6% (5). Semaglutide 2.4 mg has consequently become a widely adopted reference standard against which newer agents are judged. Despite these advances, many patients do not reach ambitious weight-loss thresholds with GLP-1 receptor agonist monotherapy, motivating combination strategies that engage complementary mechanisms of appetite and energy regulation. Amylin, a pancreatic hormone co-secreted with insulin, slows gastric emptying and promotes satiety through actions in the hindbrain and hypothalamus (6). Cagrilintide, a long-acting amylin analogue, induced dose-dependent weight loss as monotherapy in a phase 2 dose-finding trial (7), and its co-administration with semaglutide 2.4 mg, known as CagriSema, proved well tolerated with promising weight reductions in an early phase 1b study (8). A subsequent phase 2 trial in adults with type 2 diabetes reported that CagriSema produced greater weight loss than either component alone (9), and the phase 3 REDEFINE programme confirmed substantial and clinically meaningful weight reduction in adults with overweight or obesity, with and without type 2 diabetes (10–12).

Although the efficacy of CagriSema is now well established, a critical evidence gap remains. The pivotal REDEFINE 1 and REDEFINE 2 trials were designed primarily as placebo-controlled studies, and the direct comparison against semaglutide 2.4 mg monotherapy, the current standard of care, has been examined only recently and unevenly. REDEFINE 1 included a semaglutide monotherapy arm as a secondary comparison (10), and REDEFINE 5 was the first trial designed explicitly as a head-to-head comparison of CagriSema with semaglutide 2.4 mg in an east Asian population (12). This direct evidence remains dispersed across trials differing markedly in phase, treatment duration, geography, and diabetes status. Three quantitative syntheses have addressed parts of this evidence base (13–15), but each was constrained by the trials available at the time of searching, by the estimands reported in the source publications, or by the range of outcomes and certainty-of-evidence appraisal undertaken, leaving the incremental value of adding cagrilintide to semaglutide incompletely characterised. Clarifying whether the addition of cagrilintide offers incremental benefit over semaglutide alone, and at what tolerability cost, is essential to inform clinical decision-making, because patients and prescribers ultimately weigh CagriSema not against placebo but against an already effective active therapy.

We therefore conducted a systematic review and meta-analysis of randomised controlled trials that directly compared once-weekly CagriSema with semaglutide 2.4 mg monotherapy in adults with overweight or obesity, encompassing both individuals with and without type 2 diabetes, because both populations are potential candidates for combination anti-obesity pharmacotherapy in current practice. Our primary objective was to quantify the comparative effects of CagriSema versus semaglutide 2.4 mg on body weight, glycaemic, and cardiometabolic outcomes. Secondary objectives were to characterise the CagriSema-versus-placebo comparison as a contextual benchmark for the magnitude of the treatment effect, to evaluate the comparative safety and tolerability profile, and to examine treatment effects across trials enrolling participants with and without type 2 diabetes.

## Methods

### Protocol and registration

This systematic review and meta-analysis was conducted and reported in accordance with the Preferred Reporting Items for Systematic Reviews and Meta-Analyses (PRISMA) 2020 statement (16); the completed PRISMA 2020 checklist is provided as **Supplementary Table S2**. The review protocol was registered prospectively with PROSPERO (CRD420261380458).

Three changes were made to the registered protocol after registration and are declared here in full. First, the primary analysis was harmonised to the trial-product estimand for all contributing trials (see Estimands). Second, the between-study variance estimator was changed from DerSimonian–Laird to restricted maximum likelihood with a Hartung–Knapp–Sidik–Jonkman small-sample adjustment (see Statistical analysis). Third, absolute change in body weight (kg) and change in body-mass index were added as efficacy outcomes, and the required information size for the trial sequential analysis was anchored on an externally specified minimal important difference rather than on the observed pooled effect.

### Eligibility criteria

Studies were eligible if they were randomised controlled trials enrolling adults aged at least 18 years with overweight or obesity, either with concomitant type 2 diabetes or without diabetes, as prespecified in the protocol; both populations were included and analysed together, with diabetes status examined as a source of clinical heterogeneity where trial-level data permitted, that compared the co-administered or fixed-dose combination of cagrilintide and semaglutide (CagriSema, both titrated to 2.4 mg) against a comparator arm of semaglutide 2.4 mg monotherapy or placebo, with both arms receiving lifestyle intervention where applicable.

A trial in which every arm received background semaglutide 2.4 mg and randomisation determined only the addition of cagrilintide or matching placebo was treated as a CagriSema-versus-semaglutide comparison, because the randomised contrast isolates the incremental effect of cagrilintide added to semaglutide.

Eligible trials had to report at least one outcome of interest related to body weight, glycaemic control, cardiometabolic risk factors, patient-reported physical function, or safety. No restrictions were applied with respect to language, publication date, trial phase, or geographic region. Non-randomised studies, single-arm studies, observational studies, narrative reviews, and conference abstracts without an accompanying full peer-reviewed publication were excluded.

### Information sources and search strategy

A systematic literature search was performed from database inception to 30 June 2026 across four electronic databases, PubMed/MEDLINE, Embase, the Cochrane Central Register of Controlled Trials (CENTRAL), and ScienceDirect, and two international clinical trial registries: ClinicalTrials.gov and the World Health Organization International Clinical Trials Registry Platform (WHO ICTRP). The search strategy was constructed around the intervention, combining the terms CagriSema, cagrilintide, the development codes NNC0174–0833 and AM833, and amylin analogue, linked with semaglutide and glucagon-like peptide-1, using database-appropriate field tags, truncation, and controlled vocabulary. Because the intervention terms are highly specific to a recently introduced agent, population restrictions were applied only where required to preserve sensitivity. Reference lists of included trials and relevant reviews were screened manually to identify additional eligible records. The exact expression entered into each database and registry, the date each search was run, and the number of records retrieved are given in **Supplementary Table S1**.

### Study selection

All retrieved records from the four bibliographic databases and the two clinical trial registries were imported into Zotero, where duplicate records were identified and removed. Two reviewers independently screened titles and abstracts against the eligibility criteria within Zotero, after which the full texts of potentially eligible records were assessed. Disagreements were resolved by discussion or, where necessary, by consultation with a third reviewer.

### Data extraction and risk of bias assessment

Two reviewers independently extracted data using a standardised data-extraction form developed in Google Sheets, capturing trial identifiers, design and phase, country, sample size, participant characteristics, diabetes status, intervention and comparator details, follow-up duration, estimand, and outcome estimates with corresponding measures of variability. Where standard deviations were not reported, they were derived from standard errors or 95% confidence intervals using standard formulae. For outcomes reported only as arm-level means with standard deviations, the mean difference and its variance were computed directly from the arm-level data. Discrepancies were reconciled by consensus.

For every prespecified outcome, availability was recorded for each trial as reported, not reported, or not estimable, and the resulting matrix is presented in full as **Supplementary Table S3**, so that the reason each trial does or does not contribute to each pooled estimate is explicit.

The risk of bias of each included trial was assessed independently by two reviewers using the Cochrane Risk of Bias 2 (RoB 2) tool for randomised trials, evaluating the randomization process, deviations from intended interventions, missing outcome data, measurement of the outcome, and selection of the reported result (17).

Because the included trials differed markedly in treatment duration, from Enebo 2021 (20 weeks total) through Frias 2023 (32 weeks) to the 68-week REDEFINE trials (Davies 2025, Garvey 2025, Yamauchi 2026), the primary analysis pooled the end-of-treatment estimate reported by each trial, rather than a common time point. To quantify the influence of this decision, a prespecified sensitivity analysis restricted every outcome to the three 68-week phase 3a trials.

### Outcomes and effect measures

The primary outcome was the relative change in body weight, expressed as a percentage, from baseline to end of treatment.

Secondary efficacy outcomes were the absolute change in body weight (kg); the change in body-mass index; the proportion of participants achieving weight reductions of at least 5%, 10%, 15%, and 20%; changes in waist circumference, systolic blood pressure, diastolic blood pressure, glycated haemoglobin, and fasting plasma glucose; and patient-reported physical function assessed with the Impact of Weight on Quality of Life–Lite Clinical Trials Version (IWQOL-Lite-CT) Physical Function score and the Short Form 36-Item Health Survey version 2 (SF-36v2) Physical Functioning score.

Safety outcomes were any adverse events, serious adverse events, gastrointestinal adverse events, injection-site reactions, treatment discontinuation due to adverse events, and neoplasms. Continuous outcomes were analysed as mean differences using the generic inverse-variance method, with standard errors derived from reported standard errors or confidence intervals. Dichotomous outcomes were analysed as risk ratios with corresponding 95% confidence intervals. For safety outcomes, observed participant counts from each trial’s safety analysis set were used. For the categorical weight-reduction thresholds, the included trials report model-based estimated proportions rather than raw counts, except Frias 2023, which reports observed counts; estimated event numbers were therefore reconstructed as the estimated proportion multiplied by the analysis-set denominator, and observed counts were used for Frias 2023. This reconstruction is a recognised approximation that slightly understates uncertainty, and it is acknowledged in the Limitations. Outcomes with no events in either arm of a trial were not estimable and that trial was excluded from the corresponding pooled estimate; a 0.5 continuity correction was applied where events occurred in one arm only. Fasting plasma glucose was converted to mmol/L where reported in mg/dL (divisor 18.0182) so that all trials contribute on a common scale. Arm-level mean changes, measures of variability and analysis denominators for every continuous outcome are reported in **Supplementary Table S5**, responder counts for the categorical thresholds in **Supplementary Table S6**, and event counts for every safety outcome in **Supplementary Table S7**, together with the standard error used in pooling each estimate, so that every pooled result can be reproduced from the published trial reports.

### Estimands

The included trials report results under two estimands. The treatment-policy estimand assesses the effect of assignment to treatment regardless of discontinuation or rescue intervention, and the trial-product estimand assesses the effect of treatment taken as intended, without discontinuation or rescue intervention (18). Pooling estimates derived under different estimands combines answers to different clinical questions and was a source of unquantified heterogeneity in the original analysis.

Because the trial-product estimand is the only estimand reported by all five trials, Enebo 2021 and Frias 2023 report on-treatment analyses, Garvey 2025 and Davies 2025 report both estimands in their supplementary appendices, and Yamauchi 2026 designates the trial-product estimand as primary, the trial-product estimand was used for every trial in the primary analysis. The treatment-policy estimand was analysed as a prespecified sensitivity analysis for the primary outcome, for which it is numerically reported by Garvey 2025, Davies 2025 and Yamauchi 2026.

### Statistical analysis

Because the included trials used different comparators, every outcome was analysed within two prespecified comparator strata, CagriSema versus semaglutide 2.4 mg (the primary comparison) and CagriSema versus placebo (a contextual comparison). One four-arm trial (Garvey 2025) reported both a semaglutide and a placebo comparison and was therefore entered separately in each stratum; because its shared CagriSema arm contributes to both strata, the two pooled estimates are not statistically independent, a formal subgroup-interaction test between strata was not performed, and no combined cross-comparator total is reported.

Effects were pooled using a random-effects model with the between-study variance (τ²) estimated by restricted maximum likelihood (19). Confidence intervals and p-values were derived using the Hartung–Knapp–Sidik–Jonkman (HKSJ) small-sample adjustment, which replaces the normal quantile with a t quantile on k − 1 degrees of freedom and recalculates the variance of the pooled estimate from the observed dispersion of study effects (20). Where only two trials contribute to an outcome, the HKSJ interval is based on the t distribution with one degree of freedom and is so wide as to be uninterpretable. For such outcomes the REML estimate with a Wald-type interval is therefore reported as the primary result, the HKSJ interval is reported alongside it in every table, and the HKSJ interval is the interval used to judge imprecision under GRADE. This rule was fixed before the revised analyses were inspected and applies uniformly to all two-trial outcomes in both strata.

τ² is reported for every pooled estimate. Statistical heterogeneity was additionally quantified using the I² statistic and the chi-squared test. For outcomes informed by three or more trials, a 95% prediction interval is reported, computed as the pooled estimate plus or minus the t quantile on k − 2 degrees of freedom multiplied by the square root of the sum of τ² and the squared standard error of the pooled estimate (21); where the resulting interval spans values incompatible with any plausible treatment effect this is stated explicitly rather than reported as a numerical range.

Three sensitivity analyses were prespecified: leave-one-out analysis, in which each trial was sequentially removed and the analysis re-run; restriction to the three 68-week phase 3a trials; and, for the primary outcome, re-analysis under the treatment-policy estimand. Results under the DerSimonian–Laird estimator without small-sample adjustment (22) are additionally reported in **Supplementary Table S4** to allow direct comparison with previously published syntheses. Analyses were performed in Python 3.12 (NumPy 2.4, SciPy 1.17). The Python analysis code and figure-generation scripts were developed with the assistance of Claude (Anthropic; claude-opus 5), and were reviewed and verified by the authors prior to use.

### Trial sequential analysis

Trial sequential analysis (TSA) was performed for the primary outcome (percentage change in body weight) in the CagriSema-versus-semaglutide stratum, using a two-sided alpha of 5%, 80% power, and monitoring boundaries constructed with the O’Brien–Fleming alpha-spending function (23, 24).

In the original analysis the required information size (RIS) was derived from the effect size observed in the same meta-analysis, which makes the resulting inference circular. In this revision the RIS is instead anchored on an externally specified minimal important between-group difference of 3 percentage points in relative body-weight change, a threshold that is not derived from these data and that is smaller than the difference any of the included trials was powered to detect. The variance input was an outcome standard deviation of 9.0 percentage points, obtained from the arm-level standard errors reported by REDEFINE 5 (standard error 0.7 with 164 and 167 participants per arm). The RIS was adjusted for diversity (D²) computed from the random-effects and fixed-effect variances of the pooled estimate (24). Because the choice of minimal important difference is a judgement rather than an observation, the RIS is additionally tabulated for thresholds of 2 and 5 percentage points.

TSA was not performed for other outcomes, for which the small number of trials and the degree of heterogeneity make the analysis uninformative. TSA results are reported as supportive rather than confirmatory.

### Certainty of evidence

The certainty of evidence was appraised using the GRADE framework (25).

Inconsistency was judged from τ², I², the overlap of trial-level confidence intervals, and the width of the prediction interval where available, not from I² alone. Indirectness was rated as serious only for outcomes to which trials of markedly different treatment duration or trial phase contributed, and was not downgraded for outcomes informed solely by the 68-week phase 3a trials. Harmonising the estimand removed estimand heterogeneity as a source of indirectness. Imprecision was judged by comparing the confidence interval with prespecified minimal important differences and by assessing whether the optimal information size was met. The thresholds applied were 3 percentage points for relative body-weight change, 3 kg for absolute body-weight change, 1 kg/m² for body-mass index, 2 cm for waist circumference, 2 mm Hg for systolic and diastolic blood pressure, 0.3 percentage points for glycated haemoglobin, 0.5 mmol/L for fasting plasma glucose, and risk ratios of 0.80 and 1.25 for dichotomous outcomes. For the patient-reported physical-function scores no established between-group minimal important difference exists, and imprecision was judged from the interval width relative to the within-participant meaningful change thresholds used in the included trials (14.6 points for IWQOL-Lite-CT and 3.7 points for SF-36v2). For outcomes informed by only two trials, τ² is estimated with one degree of freedom; such outcomes were rated down one level for imprecision unless the HKSJ interval excluded the minimal important difference.

Publication bias could not be assessed with funnel-plot methods or regression tests, which are uninterpretable with fewer than ten trials. Rather than applying a blanket downgrade, we searched ClinicalTrials.gov and the WHO ICTRP for registered but unpublished eligible trials; no completed eligible trial lacking a full publication was identified. Publication bias was therefore recorded as undetected but not formally assessable, and was downgraded only where an outcome was measured but not reported by one or more contributing trials, which constitutes selective reporting within the available evidence. Every downgrade decision is supported by an outcome-specific explanatory footnote in **Tables 1a**, **1b**, and the number of trials contributing to each GRADE assessment matches the number shown in the corresponding forest plot.

**Table 1a:** GRADE summary of findings: CagriSema versus semaglutide 2.4 mg.

Outcome	Effect (95% CI)	k	Risk of bias	Inconsistency	Indirectness	Imprecision	Publication bias	Certainty
Percentage change in body weight	MD −6.89 (−8.70 to −5.06)	4	Not serious ¹	Not serious ²	Serious ³	Not serious ^4^	Undetected ^5^	⊕⊕⊕◯ Moderate
Absolute change in body weight	MD −7.16 kg (−9.15 to −5.17)	4	Not serious ¹	Not serious ²	Serious ³	Not serious ^4^	Undetected ^5^	⊕⊕⊕◯ Moderate
Change in body-mass index	MD −2.41 kg/m² (−2.83 to −1.99)	3	Not serious ¹	Not serious ^6^	Serious ³	Not serious ^7^	Undetected ^5^	⊕⊕⊕◯ Moderate
≥5% body-weight reduction	RR 1.14 (1.03 to 1.25)	2	Not serious ^8^	Serious ^9^	Not serious ¹^0^	Serious ¹¹	Undetected ^5^	⊕⊕◯◯ Low
≥10% body-weight reduction	RR 1.92 (0.34 to 10.78)	3	Not serious ^8^	Very serious ¹²	Serious ³	Very serious ¹³	Undetected ^5^	⊕◯◯◯ Very low
≥15% body-weight reduction	RR 1.76 (0.68 to 4.58)	3	Not serious ^8^	Serious ¹²	Serious ³	Serious ¹³	Undetected ^5^	⊕◯◯◯ Very low
≥20% body-weight reduction	RR 2.10 (1.78 to 2.47)	2	Not serious ^8^	Not serious ¹^4^	Not serious ¹^0^	Not serious ¹^5^	Undetected ^5^	⊕⊕⊕⊕ High
Change in waist circumference	MD −4.07 cm (−5.10 to −3.04)	2	Not serious ^8^	Not serious ¹^4^	Not serious ¹^0^	Serious ¹^6^	Undetected ^5^	⊕⊕⊕◯ Moderate
Change in systolic blood pressure	MD −3.21 mm Hg (−5.90 to −0.53)	2	Not serious ^8^	Serious ¹^7^	Not serious ¹^0^	Serious ¹^8^	Undetected ^5^	⊕⊕◯◯ Low
Change in diastolic blood pressure	MD −1.95 mm Hg (−5.38 to 1.47)	2	Not serious ^8^	Very serious ¹^9^	Not serious ¹^0^	Serious ²^0^	Undetected ^5^	⊕◯◯◯ Very low
Change in glycated haemoglobin	MD −0.07 (−0.14 to 0.01)	4	Not serious ¹	Not serious ²	Serious ²¹	Not serious ²²	Undetected ^5^	⊕⊕⊕◯ Moderate
Change in fasting plasma glucose	MD −0.07 mmol/L (−0.23 to 0.08)	4	Not serious ¹	Not serious ²	Serious ²¹	Not serious ²²	Undetected ^5^	⊕⊕⊕◯ Moderate
IWQOL-Lite-CT Physical Function	MD 1.40 (−2.00 to 4.80)	1	Serious ²³	Not assessable ²^4^	Serious ²^5^	Serious ²^6^	Serious ²^7^	⊕◯◯◯ Very low
SF-36v2 Physical Functioning	MD −0.30 (−1.20 to 0.60)	1	Serious ²³	Not assessable ²^4^	Serious ²^5^	Serious ²^6^	Serious ²^7^	⊕◯◯◯ Very low
Any adverse event	RR 1.03 (1.01 to 1.05)	4	Not serious ¹	Not serious ²^8^	Serious ²^9^	Not serious ³^0^	Undetected ^5^	⊕⊕⊕◯ Moderate
Serious adverse events	RR 0.91 (0.08 to 10.80)	3	Not serious ^8^	Serious ³¹	Serious ²^9^	Very serious ³²	Undetected ^5^	⊕◯◯◯ Very low
Gastrointestinal adverse events	RR 1.09 (0.97 to 1.21)	4	Not serious ¹	Not serious ²^8^	Serious ²^9^	Not serious ³³	Undetected ^5^	⊕⊕⊕◯ Moderate
Discontinuation due to adverse events	RR 1.35 (0.41 to 4.51)	3	Not serious ^8^	Not serious ³^4^	Serious ²^9^	Very serious ³²	Undetected ^5^	⊕◯◯◯ Very low
Injection-site reactions	RR 3.14 (1.42 to 6.96)	4	Not serious ¹	Serious ³^5^	Serious ²^9^	Not serious ³^6^	Undetected ^5^	⊕⊕◯◯ Low
Neoplasms	RR 1.02 (0.68 to 1.52)	2	Not serious ^8^	Not serious ¹^4^	Serious ³^7^	Very serious ³^8^	Undetected ^5^	⊕◯◯◯ Very low

*Inconsistency* was judged from τ², I², the overlap of the trial-level confidence intervals, and the width of the 95% prediction interval where three or more trials contributed; it was not judged from I² alone.

*Indirectness* was rated serious only where trials of markedly different treatment duration or trial phase contributed to the estimate, and was not downgraded where an estimate was informed solely by the 68-week phase 3a trials.

*Imprecision* was judged by comparing the confidence interval with a prespecified minimal important difference and by assessing whether the optimal information size was met. The thresholds were 3 percentage points for relative body-weight change, 3 kg for absolute body-weight change, 1 kg/m² for body-mass index, 2 cm for waist circumference, 2 mm Hg for systolic and diastolic blood pressure, 0.3 percentage points for glycated haemoglobin, 0.5 mmol/L for fasting plasma glucose, and risk ratios of 0.80 and 1.25 for dichotomous outcomes. Three consequences follow. First, an interval lying entirely beyond the minimal important difference is precise and is not rated down. Second, an interval lying entirely within the minimal important difference is also precise — it is a precise null — and is not rated down. Third, for outcomes informed by only two trials, where τ² is estimated with a single degree of freedom, imprecision was rated down one level unless the Hartung–Knapp–Sidik–Jonkman interval excluded the minimal important difference.

*Publication bias* could not be assessed by funnel-plot or regression methods, which are uninterpretable with fewer than ten trials. It was recorded as undetected but not formally assessable, and was rated down only where an outcome was measured but not reported by a contributing trial.

¹ Three of the four contributing trials are at low overall risk of bias. Enebo 2021 raises some concerns for selection of the reported result but carries between 5.7% and 15.9% of the weight depending on outcome, and the leave-one-out estimate omitting it does not differ materially (**Supplementary Figure S3**).

² τ² = 0.000 and I² ≤ 38%; the trial-level confidence intervals overlap and the 95% prediction interval lies entirely on the side of benefit.

³ Contributing trials differ in treatment duration (20 weeks in Enebo 2021, 32 weeks in Frias 2023, 68 weeks in the phase 3a trials) and in trial phase (1b to 3a). Because weight loss accrues progressively, pooling end-of-treatment estimates across these durations conflates the magnitude of effect with its temporal maturity. Rated down one level.

^4^ The entire confidence interval lies beyond the minimal important difference of 3 percentage points (3 kg for absolute weight change), and the optimal information size is met: 2,835 participants accrued against a diversity-adjusted required information size of 587 (**Figure 6**).

^5^ Funnel-plot asymmetry and regression tests are uninterpretable with fewer than ten trials. Searching ClinicalTrials.gov and the WHO ICTRP identified no completed eligible trial without a full publication, and the outcome is reported by every trial that measured it. Common sponsorship of all included trials by a single manufacturer is noted in the Limitations but does not by itself establish selective non-publication for this outcome.

^6^ τ² = 0.000 and I² = 0%; the trial-level intervals overlap closely. The width of the prediction interval (−4.72 to −0.10) reflects the t-multiplier applied with only three contributing trials rather than observed heterogeneity, and did not prompt a downgrade.

^7^ The confidence interval (−2.83 to −1.99) lies entirely beyond the minimal important difference of 1 kg/m².

^8^ All trials contributing to this estimate are at low overall risk of bias.

^9^ I² = 78% with only two contributing trials, and the trial-level risk ratios (1.09 and 1.20) do not overlap. Rated down one level.

¹^0^ Both contributing trials are 68-week phase 3a trials reporting the same estimand, so no indirectness downgrade applies.

¹¹ Only two trials contribute, so τ² is estimated with one degree of freedom, and the HKSJ interval (0.60 to 2.15) includes both appreciable benefit and appreciable harm. Rated down one level.

¹² Frias 2023, a 62-participant phase 2 trial in type 2 diabetes with a very low comparator response (4 of 29 and 0 of 29 participants), yields risk ratios of 5.18 and 32.07 that are incompatible in scale with those from the phase 3a trials (1.21–1.66 and 1.54–2.00). I² = 90% for the ≥10% threshold and 74% for the ≥15% threshold. Rated down two levels for ≥10% and one level for ≥15%, where the phase 3a trials agree more closely.

¹³ The confidence interval spans the null and includes both appreciable benefit and appreciable harm; for the ≥10% threshold the prediction interval is uninformative. Rated down two levels for ≥10% and one level for ≥15%.

¹^4^ τ² = 0.000, I² = 0%, and the trial-level confidence intervals overlap.

¹^5^ Although only two trials contribute, the HKSJ interval (1.74 to 2.54) lies entirely beyond the 1.25 threshold for an appreciable effect, and 2,741 participants contribute with abundant events. No downgrade applied.

¹^6^ The HKSJ interval extends to −1.69 cm, which is smaller than the minimal important difference of 2 cm, so a difference below the threshold of clinical importance is not excluded. Rated down one level.

¹^7^ The two contributing trials report −2.1 mm Hg (Garvey 2025) and −4.9 mm Hg (Yamauchi 2026), with τ² = 2.61, I² = 67% and non-overlapping confidence intervals. Rated down one level.

¹^8^ The HKSJ interval (−20.63 to 14.20) crosses the null and spans values on both sides of the 2 mm Hg threshold. Rated down one level.

¹^9^ τ² = 5.53 and I² = 90%: Garvey 2025 reports −0.3 mm Hg with an interval crossing the null, whereas Yamauchi 2026 reports −3.8 mm Hg with an interval excluding it. Rated down two levels.

²^0^ The confidence interval (−5.38 to 1.47) crosses the null and includes differences on both sides of the 2 mm Hg threshold. Rated down one level.

²¹ Contributing trials differ in duration and, critically, in baseline glycaemic status: Frias 2023 and part of Yamauchi 2026 enrolled participants with type 2 diabetes, whereas Garvey 2025 excluded diabetes and Enebo 2021 enrolled metabolically healthy volunteers. Rated down one level.

²² The entire confidence interval lies within the minimal important difference (0.3 percentage points for glycated haemoglobin, 0.5 mmol/L for fasting plasma glucose), so this is a precise null rather than an inconclusive result. No downgrade applied.

²³ The only contributing trial (Yamauchi 2026) is at some concerns for missing outcome data, with imbalanced premature discontinuation (17 of 164 versus 10 of 167). Rated down one level.

²^4^ Consistency cannot be assessed from a single trial.

²^5^ The only contributing trial was conducted exclusively in Japan and Taiwan, so the estimate may not transport to the predominantly White populations of the other trials. Rated down one level.

²^6^ The estimate derives from a single 331-participant trial and the optimal information size is not met, although the confidence interval excludes the within-participant meaningful change thresholds used in the trials (14.6 points for IWQOL-Lite-CT and 3.7 points for SF-36v2). Rated down one level.

²^7^ Garvey 2025 measured both instruments but reports them only against placebo, so a head-to-head estimate that exists in the trial data is not available in the public record. This is selective reporting of an available outcome within the included evidence. Rated down one level.

²^8^ τ² = 0.000 and I² ≤ 7%; the trial-level intervals overlap and the prediction interval lies within the prespecified thresholds.

²^9^ Risk ratios are pooled across exposure periods of 20, 32 and 68 weeks and are therefore not equivalent to incidence-rate comparisons, because adverse-event risk accrues with exposure. Rated down one level.

³^0^ The confidence interval (1.01 to 1.05) lies entirely within the prespecified range of 0.80 to 1.25, and 2,837 participants contribute. No downgrade applied.

³¹ Garvey 2025 reports a risk ratio of 1.97 favouring semaglutide while Yamauchi 2026 reports 0.56 in the opposite direction, with τ² = 0.66 and I² = 71%. Rated down one level.

³² The confidence interval is compatible with both a substantial reduction and a substantial increase in risk (0.08 to 10.80 for serious adverse events; 0.41 to 4.51 for discontinuation). Events are sparse outside the largest trial (212 of 2,301 versus 28 of 500, and 128 of 2,301 versus 16 of 500 respectively) and the optimal information size is far from met. Rated down two levels.

³³ The confidence interval (0.97 to 1.21) lies entirely within the prespecified range of 0.80 to 1.25, and the information size is met (1,792 events among 2,313 participants receiving CagriSema versus 337 among 524 receiving semaglutide). No downgrade applied. Because this outcome is common, a risk ratio of 1.21 at the upper bound corresponds to an absolute difference of approximately 13 percentage points; the rating reflects precision on the relative scale, on which the thresholds were prespecified.

³^4^ τ² = 0.031 and I² = 0%, and the trial-level intervals overlap. The uninformative prediction interval reflects the small number of contributing trials and is captured under imprecision rather than inconsistency.

³^5^ Although I² is 8%, τ² is 0.111 and the 95% prediction interval (0.44 to 22.29) crosses the null, indicating that the effect in a future trial is not confidently predictable from these four trials. Rated down one level.

³^6^ The confidence interval (1.42 to 6.96) lies entirely beyond the 1.25 threshold, and 297 events contribute. No downgrade applied.

³^7^ Maximum follow-up is 68 weeks, far shorter than the latency of most malignancies, so the outcome as measured is an indirect surrogate for the clinical question of neoplasm risk. Rated down one level.

³^8^ Only two trials contribute, events are sparse (145 among 2,270 participants receiving CagriSema versus 29 among 469 receiving semaglutide), and the confidence interval (0.68 to 1.52) includes both appreciable benefit and appreciable harm. Rated down two levels. Absence of a statistically significant difference does not establish equivalence.

**Table 1b:** GRADE summary of findings: CagriSema versus placebo.

Outcome	Effect (95% CI)	k	Risk of bias	Inconsistency	Indirectness	Imprecision	Publication bias	Certainty
Percentage change in body weight	MD −16.50 (−24.15 to −8.86)	2	Not serious ¹	Very serious ²	Not serious ³	Serious ^4^	Undetected ^5^	⊕◯◯◯ Very low
Absolute change in body weight	MD −17.30 kg (−25.73 to −8.88)	2	Not serious ¹	Very serious ²	Not serious ³	Serious ^4^	Undetected ^5^	⊕◯◯◯ Very low
Change in body-mass index	MD −6.20 kg/m² (−9.14 to −3.26)	2	Not serious ¹	Very serious ²	Not serious ³	Serious ^4^	Undetected ^5^	⊕◯◯◯ Very low
≥5% body-weight reduction	RR 3.29 (2.77 to 3.90)	2	Not serious ¹	Serious ^6^	Not serious ³	Serious ^7^	Undetected ^5^	⊕⊕◯◯ Low
≥10% body-weight reduction	RR 7.92 (6.63 to 9.46)	2	Not serious ¹	Not serious ^8^	Not serious ³	Not serious ^9^	Undetected ^5^	⊕⊕⊕⊕ High
≥15% body-weight reduction	RR 19.93 (11.53 to 34.44)	2	Not serious ¹	Not serious ¹^0^	Not serious ³	Serious ¹¹	Undetected ^5^	⊕⊕⊕◯ Moderate
≥20% body-weight reduction	RR 37.91 (22.09 to 65.07)	2	Not serious ¹	Not serious ^8^	Not serious ³	Not serious ¹²	Undetected ^5^	⊕⊕⊕⊕ High
Change in waist circumference	MD −12.75 cm (−17.95 to −7.56)	2	Not serious ¹	Very serious ²	Not serious ³	Serious ^4^	Undetected ^5^	⊕◯◯◯ Very low
Change in systolic blood pressure	MD −7.18 mm Hg (−10.50 to −3.85)	2	Not serious ¹	Serious ¹³	Not serious ³	Serious ^4^	Undetected ^5^	⊕⊕◯◯ Low
Change in diastolic blood pressure	MD −2.38 mm Hg (−5.42 to 0.66)	2	Not serious ¹	Very serious ¹^4^	Not serious ³	Serious ^4^	Undetected ^5^	⊕◯◯◯ Very low
Change in glycated haemoglobin	MD −1.22 (−2.75 to 0.31)	2	Not serious ¹	Very serious ¹^5^	Not serious ³	Serious ^4^	Undetected ^5^	⊕◯◯◯ Very low
Change in fasting plasma glucose	MD −1.77 mmol/L (−4.17 to 0.64)	2	Not serious ¹	Very serious ¹^5^	Not serious ³	Serious ^4^	Undetected ^5^	⊕◯◯◯ Very low
IWQOL-Lite-CT Physical Function	MD 9.41 (2.65 to 16.17)	2	Not serious ¹	Very serious ¹^6^	Not serious ³	Serious ^4^	Undetected ^5^	⊕◯◯◯ Very low
SF-36v2 Physical Functioning	MD 3.15 (1.39 to 4.91)	2	Not serious ¹	Serious ¹^7^	Not serious ³	Serious ^4^	Undetected ^5^	⊕⊕◯◯ Low
Any adverse event	RR 1.09 (1.03 to 1.16)	2	Not serious ¹	Serious ¹^8^	Not serious ³	Serious ^4^	Undetected ^5^	⊕⊕◯◯ Low
Serious adverse events	RR 1.14 (0.58 to 2.24)	2	Not serious ¹	Serious ¹^9^	Not serious ³	Very serious ²^0^	Undetected ^5^	⊕◯◯◯ Very low
Gastrointestinal adverse events	RR 2.02 (1.87 to 2.19)	2	Not serious ¹	Not serious ^8^	Not serious ³	Not serious ^9^	Undetected ^5^	⊕⊕⊕⊕ High
Discontinuation due to adverse events	RR 2.02 (1.24 to 3.31)	2	Not serious ¹	Not serious ¹^0^	Not serious ³	Serious ¹¹	Undetected ^5^	⊕⊕⊕◯ Moderate
Injection-site reactions	RR 6.72 (1.24 to 36.41)	2	Not serious ¹	Not serious ¹^0^	Not serious ³	Very serious ²¹	Undetected ^5^	⊕⊕◯◯ Low
Neoplasms	RR 1.28 (0.95 to 1.73)	2	Not serious ¹	Not serious ^8^	Serious ²²	Very serious ²³	Undetected ^5^	⊕◯◯◯ Very low

¹ Both contributing trials (Davies 2025 and Garvey 2025) are at low overall risk of bias in every RoB 2 domain.

² The two contributing trials enrolled fundamentally different populations — REDEFINE 1 excluded diabetes and REDEFINE 2 required it — and produce non-overlapping estimates with I² of 99–100%. Rated down two levels.

³ Both contributing trials are 68-week phase 3a trials reporting the same estimand.

^4^ Only two trials contribute, τ² is estimated with one degree of freedom, and the HKSJ interval crosses the null for this outcome. Rated down one level.

^5^ As footnote 5 to **Table 1a**.

^6^ I² = 62% with only two contributing trials, and the trial-level risk ratios (2.98 and 3.55) do not overlap. Rated down one level.

^7^ The HKSJ interval (1.09 to 9.89) includes values below the 1.25 threshold for an appreciable effect, so an appreciable effect is not excluded. Rated down one level.

^8^ τ² ≤ 0.001, I² ≤ 2%, and the trial-level confidence intervals overlap.

^9^ The HKSJ interval lies entirely beyond the 1.25 threshold (5.71 to 10.98 for the ≥10% threshold; 1.52 to 2.70 for gastrointestinal adverse events), with abundant events across 4,019 participants. No downgrade applied.

¹^0^ I² ≤ 48% with overlapping trial-level confidence intervals.

¹¹ Although the primary confidence interval is narrow, the HKSJ interval is very wide because τ² is estimated from only two trials. Rated down one level.

¹² Although only two trials contribute, the HKSJ interval (1.75 to 822.60) lies entirely beyond the 1.25 threshold, and the event count is large (1,533 responders among 3,012 participants receiving CagriSema versus 13 among 1,007 receiving placebo). No downgrade applied.

¹³ I² = 91%: Davies 2025 reports −5.4 mm Hg and Garvey 2025 −8.8 mm Hg, with non-overlapping intervals. Rated down one level.

¹^4^ I² = 95%: Davies 2025 reports −0.8 mm Hg with an interval crossing the null and Garvey 2025 −3.9 mm Hg with an interval excluding it. Rated down two levels.

¹^5^ The pooled estimate combines a trial in participants with type 2 diabetes and baseline glycated haemoglobin of 8.0% with a trial that excluded diabetes and had baseline glycated haemoglobin of 5.5%; I² is 99–100% and the pooled value describes neither population. Rated down two levels.

¹^6^ I² = 94%, with estimates of 5.9 and 12.8 points and non-overlapping intervals. Rated down two levels.

¹^7^ I² = 88%, with estimates of 2.2 and 4.0 points. Rated down one level.

¹^8^ I² = 72%, with estimates of 1.06 and 1.12 and non-overlapping intervals. Rated down one level.

¹^9^ Davies 2025 reports a risk ratio of 0.81 and Garvey 2025 a risk ratio of 1.60, in opposite directions, with I² = 88%. Rated down one level.

²^0^ The confidence interval (0.58 to 2.24) is compatible with both appreciable benefit and appreciable harm, and the HKSJ interval is uninterpretably wide. Rated down two levels.

²¹ Injection-site reactions did not occur in the placebo arm of Davies 2025, so a continuity correction was required, and the HKSJ interval is uninterpretably wide. Rated down two levels.

²² Maximum follow-up is 68 weeks, far shorter than the latency of most malignancies. Rated down one level.

²³ Events are sparse and the confidence interval (0.95 to 1.73) includes both no effect and an appreciable increase in risk. Rated down two levels. Absence of a statistically significant difference does not establish equivalence.

## Results

### Study selection

The searches identified 1,760 records in total: 1,551 from the four bibliographic databases (PubMed/MEDLINE 162, Embase 440, Cochrane CENTRAL 96, ScienceDirect 853) and 209 from the two clinical trial registries (ClinicalTrials.gov 131, WHO ICTRP 78). All registry records corresponded to trials already retrievable from the bibliographic databases and did not yield any additional eligible trials. After removal of duplicate records in Zotero, the remaining records were screened by title and abstract, and the full texts of those judged potentially eligible were assessed against the eligibility criteria. Following full-text review, five randomised controlled trials (8–12) met all inclusion criteria and were incorporated into the quantitative synthesis. Across all five trials, 4,746 unique participants were included in the review: 2,835 contributed to the prespecified CagriSema-versus-semaglutide 2.4 mg comparison (Enebo 2021, Frias 2023, the CagriSema and semaglutide arms of Garvey 2025, and Yamauchi 2026) and 4,019 contributed to the CagriSema-versus-placebo comparison (Davies 2025 and the CagriSema and placebo arms of Garvey 2025); the CagriSema arm of Garvey 2025 (n = 2,108) contributed to both strata, and its participants are therefore counted once in the overall unique total. The full selection process, including the numbers of records excluded at each stage and the reasons for full-text exclusion, is detailed in the PRISMA 2020 flow diagram (**Figure 1**).

**Figure 1:**
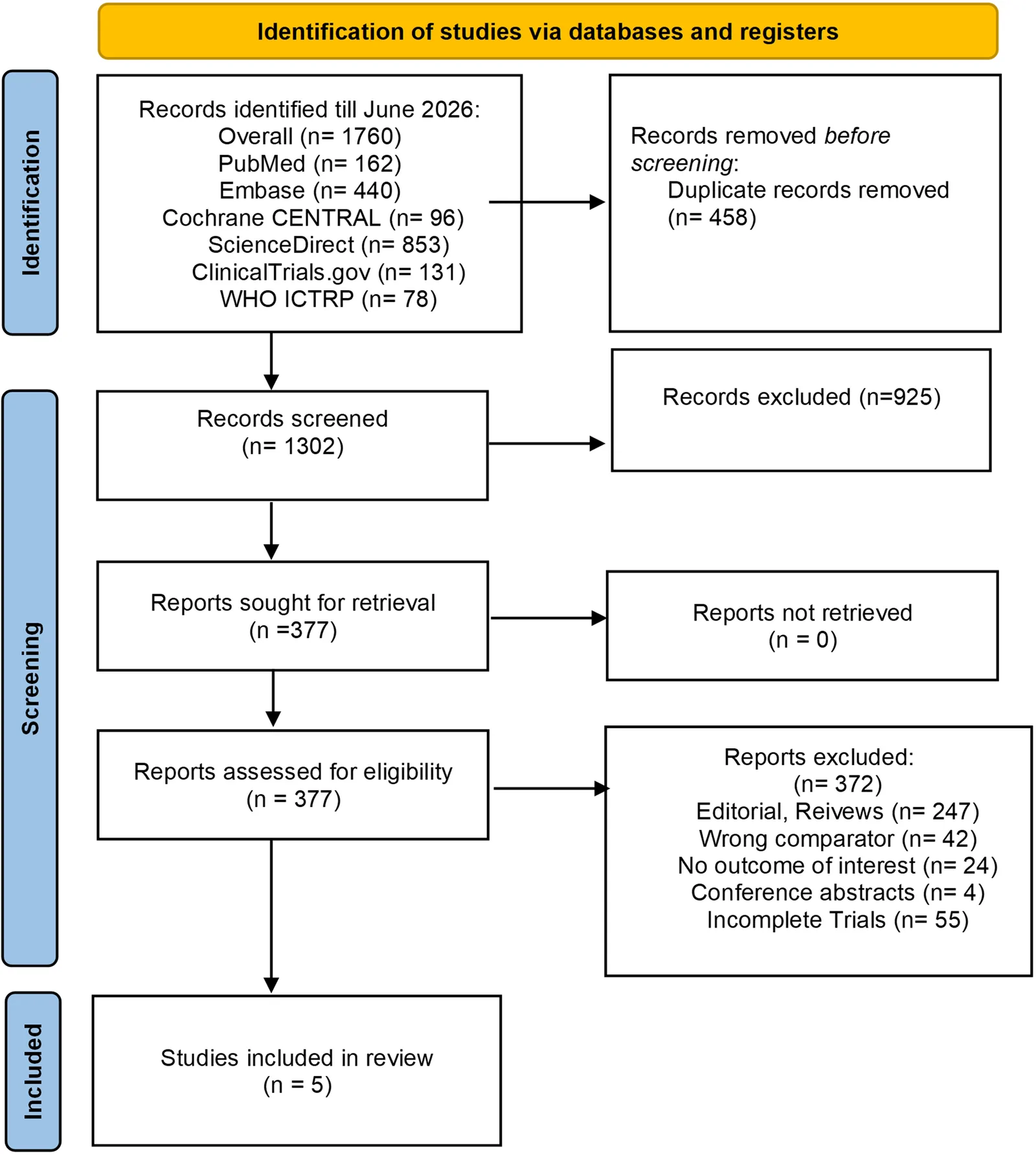
PRISMA 2020 flow diagram of study selection. Searches were run from database inception to 30 June 2026 across four bibliographic databases and two clinical trial registries. Registry records corresponded to trials already retrieved from the bibliographic databases and yielded no additional eligible trials.

### Characteristics of included studies

The five included trials enrolled participants with overweight or obesity, with and without type 2 diabetes, and spanned phase 1b through phase 3a designs conducted across single-country and multinational settings. Four trials contributed a comparison of CagriSema with semaglutide 2.4 mg (Enebo 2021, Frias 2023, Garvey 2025 and Yamauchi 2026) and two contributed a comparison with placebo (Davies 2025 and Garvey 2025); the four-arm trial (Garvey 2025) contributed both. In Enebo 2021 every arm received background semaglutide 2.4 mg and participants were randomised to cagrilintide or matching placebo, so the randomised contrast is the incremental effect of adding cagrilintide to semaglutide. Treatment duration ranged from 20 weeks to 68 weeks. Baseline weight, body-mass index, and cardiometabolic characteristics were broadly comparable between arms within each trial, while glycated haemoglobin differed by population, reflecting the inclusion of both type 2 diabetes cohorts and cohorts without diabetes. The baseline characteristics, intervention schedules, comparator definitions, and diabetes status of each trial are summarised in **Table 2**. Outcome availability by trial is given in **Supplementary Table S3**.

**Table 2:** Baseline characteristics of the included trials.

Characteristic	Davies 2025 (11) CagriSema	Davies 2025 Placebo	Enebo 2021 (8) CagriSema	Enebo 2021 Placebo + Semaglutide 2.4 mg	Frias 2023 (9) CagriSema	Frias 2023 Semaglutide	Garvey 2025 (10) CagriSema	Garvey 2025 Semaglutide	Garvey 2025 Placebo	Yamauchi 2026 (12) CagriSema	Yamauchi 2026 Semaglutide
Diabetes status	Type 2 diabetes	Type 2 diabetes	No diabetes	No diabetes	Type 2 diabetes	Type 2 diabetes	No diabetes	No diabetes	No diabetes	With/without T2D	With/without T2D
Phase of trial	Phase 3a	—	Phase 1b	—	Phase 2	—	Phase 3a	—	—	Phase 3a	—
Starting dose	0.25 mg	—	0.16 mg	—	0.25 mg	—	0.25 mg	—	—	0.25 mg	—
Escalation interval	Every 4 weeks	—	Every 4 weeks	—	Every 4 weeks	—	Every 4 weeks	—	—	Every 4 weeks	—
Target dose	2.4 mg	—	2.4 mg	—	2.4 mg	2.4 mg	2.4 mg	2.4 mg	—	2.4 mg	2.4 mg
Time to target dose	16 weeks	—	16 weeks	—	16 weeks	—	16 weeks	—	—	16 weeks	—
Total treatment duration	68 weeks	—	20 weeks	—	32 weeks	—	68 weeks	—	—	68 weeks	—
Sample size (n)	904	302	12	20	31	31	2108	302	705	164	167
Age, years (mean)	55.9	56.5	43.0	41.0	56	57	47.1	47.4	46.7	50.9	51.4
Female, n (%)	429 (47.5)	140 (46.4)	7 (58)	7 (35)	13 (42)	13 (42)	1441 (68.4)	199 (65.9)	461 (65.4)	58 (35)	47 (28)
Male, n (%)	475 (52.5)	162 (53.6)	5 (42)	13 (65)	18 (58)	18 (58)	667 (31.6)	103 (34.1)	244 (34.6)	106 (65)	120 (72)
Body weight, kg (mean)	101.9	103.3	92.1	99.6	104.3	105.4	107.2	105.9	106.6	95.2	95.4
BMI, kg/m² (mean)	36.1	36.4	32.2	32.2	35.9	36.2	38.0	37.4	37.8	34.2	34.0
Waist circumference, cm	115.6	116.4	NR	NR	NR	NR	115.0	114.2	114.1	111.0	110.8
HbA1c, % (mean)	8.0	8.0	5.2	5.4	8.5	8.6	5.5	5.5	5.5	6.2	6.3
IWQOL-Lite-CT score (mean)	59.0	59.7	NR	NR	NR	NR	54.0	55.2	54.7	64.0	62.5
SF-36v2 Physical Functioning	44.8	44.6	NR	NR	NR	NR	45.7	46.6	45.7	51.9	51.0
Systolic BP, mm Hg (mean)	130.2	130.2	121	131	130	128	126.8	127.0	128.1	129.0	127.8
Diastolic BP, mm Hg (mean)	80.4	80.4	79	86	80	79	82.0	81.8	82.5	83.1	83.1
LDL cholesterol, mmol/L	2.11	2.16	3.2	3.4	1.8	2.3	2.90	2.77	2.87	2.7	2.7
HDL cholesterol, mmol/L	1.12	1.13	1.4	1.3	1.0	1.1	1.26	1.26	1.26	1.3	1.2

CagriSema, co-administered cagrilintide and semaglutide; BMI, body-mass index; HbA1c, glycated haemoglobin; IWQOL-Lite-CT, Impact of Weight on Quality of Life–Lite Clinical Trials Version; SF-36v2, Short Form 36-Item Health Survey version 2; BP, blood pressure; LDL, low-density lipoprotein; HDL, high-density lipoprotein; NR, not reported; T2D, type 2 diabetes.

Garvey 2025 was a four-group trial. Its CagriSema arm contributes to both comparator strata, its semaglutide arm to the CagriSema-versus-semaglutide stratum, and its placebo arm to the CagriSema-versus-placebo stratum. The cagrilintide-alone arm of that trial was not eligible for either comparison and is not shown. Lipid values for Garvey 2025 are converted from mg/dL.

In Enebo 2021 all participants, in every arm, received background semaglutide 2.4 mg; randomisation determined only the addition of cagrilintide or matching placebo, so the comparator column represents semaglutide 2.4 mg monotherapy. Baseline characteristics for that comparator are those of the pooled placebo group; the analysis denominator of 20 refers to the cohort 1–5 pool used for the body-weight analyses.

All efficacy estimates in this review are taken under the trial-product estimand.

### Risk of bias

Three trials (Frias 2023, Garvey 2025, Davies 2025) were judged at low overall risk of bias. Two trials raised some concerns: Enebo 2021, because body weight was an exploratory pharmacodynamic endpoint in a phase 1b trial designed for safety and pharmacokinetics, with no prespecified hypothesis testing, and because of differential discontinuation across small cohorts; and Yamauchi 2026, because of imbalanced premature treatment discontinuation (17 of 164 with cagrilintide–semaglutide versus 10 of 167 with semaglutide) in a comparatively small sample, notwithstanding appropriate multiple imputation. No trial was judged at high risk in any domain. Domain-level judgements and overall ratings are summarised in **Figure 2**.

**Figure 2:**
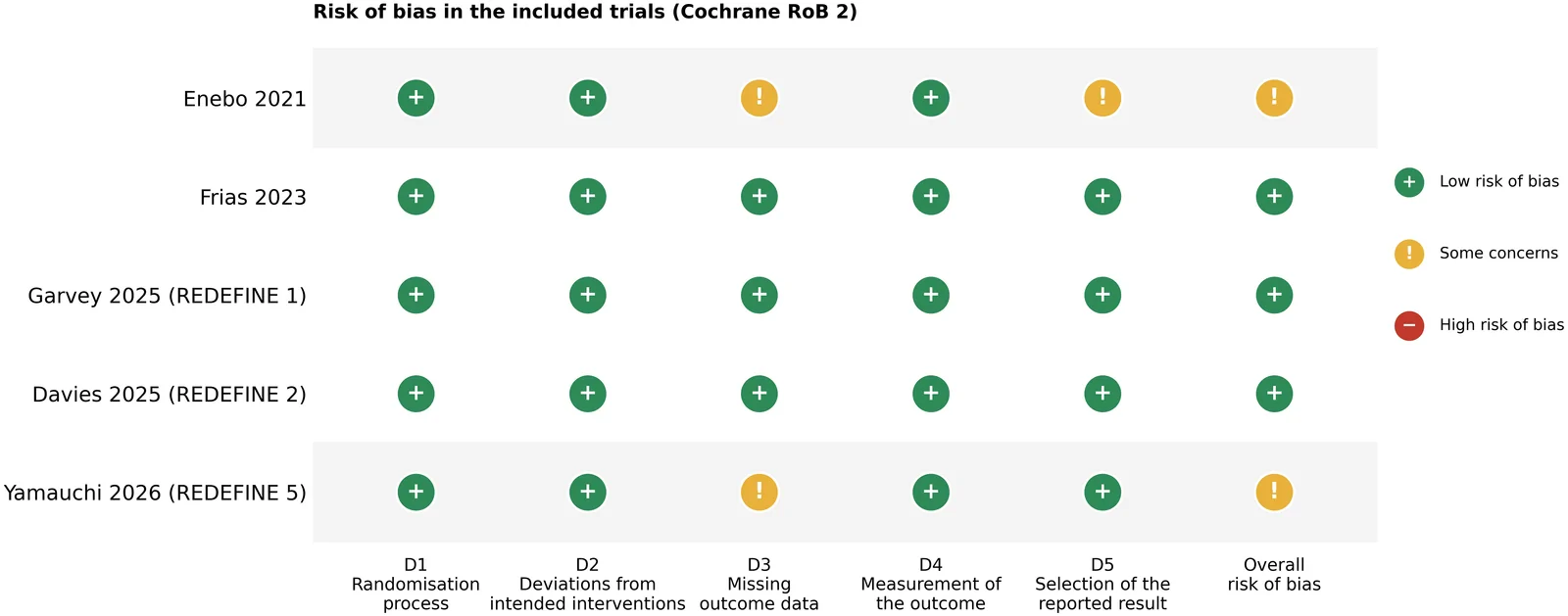
Risk of bias in the five included trials, assessed with the cochrane risk of bias 2 (RoB 2) tool across five domains and an overall judgement. Green with a plus sign denotes low risk of bias and amber with an exclamation mark denotes some concerns; no trial was judged at high risk in any domain.

### Primary outcome: percentage change in body weight

Under the harmonised trial-product estimand, CagriSema produced a significantly greater relative reduction in body weight than semaglutide 2.4 mg (**Figure 3A**). The pooled mean difference across four trials was −6.89 percentage points (95% CI −8.70 to −5.06; p = 0.001), with τ² = 0.00 and moderate heterogeneity (I² = 33%; Q p = 0.22). The 95% prediction interval, −8.91 to −4.86 percentage points, lies entirely on the side of benefit, indicating that a future trial conducted in a comparable population would be expected to show an incremental reduction of at least approximately 5 percentage points. Heterogeneity was substantially lower than in the analysis that pooled mixed estimands (I² = 60%), indicating that estimand heterogeneity, rather than clinical diversity alone, accounted for much of the previously observed inconsistency.

**Figure 3:**
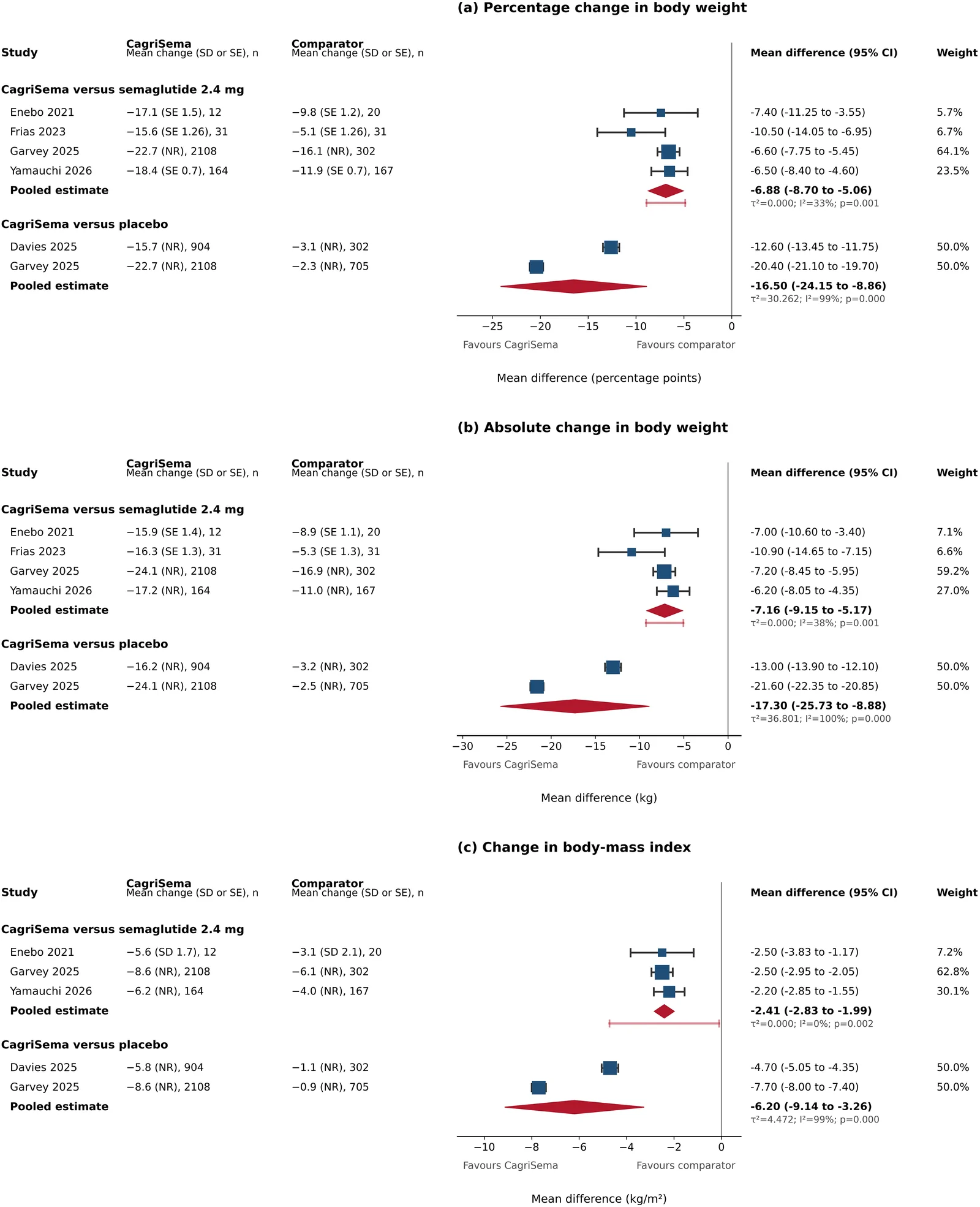
Forest plots of continuous body-weight outcomes for CagriSema versus semaglutide 2.4 mg and versus placebo, under the trial-product estimand. **(A)** Percentage change in body weight. **(B)** Absolute change in body weight (kg). **(C)** Change in body-mass index (kg/m²). Squares represent trial-level estimates, sized in proportion to weight; horizontal lines are 95% confidence intervals; diamonds are pooled random-effects estimates obtained by restricted maximum likelihood; the horizontal bar beneath a diamond, where shown, is the 95% prediction interval. τ², I² and the p-value for the pooled estimate are given beneath each diamond. Arm-level mean changes, measures of variability, analysis denominators and the standard errors used in pooling are given for every trial in **Supplementary Table S5**. Columns to the left of each plot give the estimated mean change from baseline in each arm with its reported measure of variability, labelled SD or SE, and the analysis denominator; NR denotes that the trial reported no arm-level measure of variability, in which case the standard error used in pooling was derived from the published confidence interval around the treatment difference.

Findings were robust across all prespecified sensitivity analyses. Leave-one-out estimates ranged from −6.62 to −7.82 percentage points and remained statistically significant throughout (**Supplementary Figure S3a**). Restriction to the two 68-week phase 3a trials contributing to this stratum gave −6.57 percentage points (95% CI −7.56 to −5.59; I² = 0%). Under the treatment-policy estimand, for which Garvey 2025 and Yamauchi 2026 report numerical estimates, the pooled difference was smaller but consistent in direction, at −5.58 percentage points (95% CI −6.61 to −4.55; I² = 0%), as expected because that estimand incorporates the effect of treatment discontinuation.

Versus placebo, the reduction was larger, at −16.50 percentage points (95% CI −24.15 to −8.86), but with extreme heterogeneity between the two contributing trials (τ² = 30.26; I² = 99%), reflecting the different populations enrolled by REDEFINE 1 and REDEFINE 2; the corresponding HKSJ interval (−66.06 to 33.05) is uninformative and this pooled estimate should be read as a descriptive benchmark rather than as a precise effect estimate.

### Absolute body weight and body-mass index

CagriSema produced a greater absolute reduction in body weight than semaglutide 2.4 mg, with a pooled mean difference of −7.16 kg (95% CI −9.15 to −5.17; I² = 38%; prediction interval −9.27 to −5.05) (**Figure 3B**), and a greater reduction in body-mass index, at −2.41 kg/m² (95% CI −2.83 to −1.99; I² = 0%) (**Figure 3C**). Versus placebo the corresponding differences were −17.30 kg (95% CI −25.73 to −8.88) and −6.20 kg/m² (95% CI −9.14 to −3.26), again with heterogeneity approaching 100%.

### Categorical weight-reduction responders

Estimates for the categorical thresholds are shown in **Figure 4**. Versus semaglutide 2.4 mg, the pooled risk ratio was 1.14 (95% CI 1.03 to 1.25) for a reduction of at least 5% and 2.10 (95% CI 1.78 to 2.47; I² = 0%) for a reduction of at least 20%.

**Figure 4:**
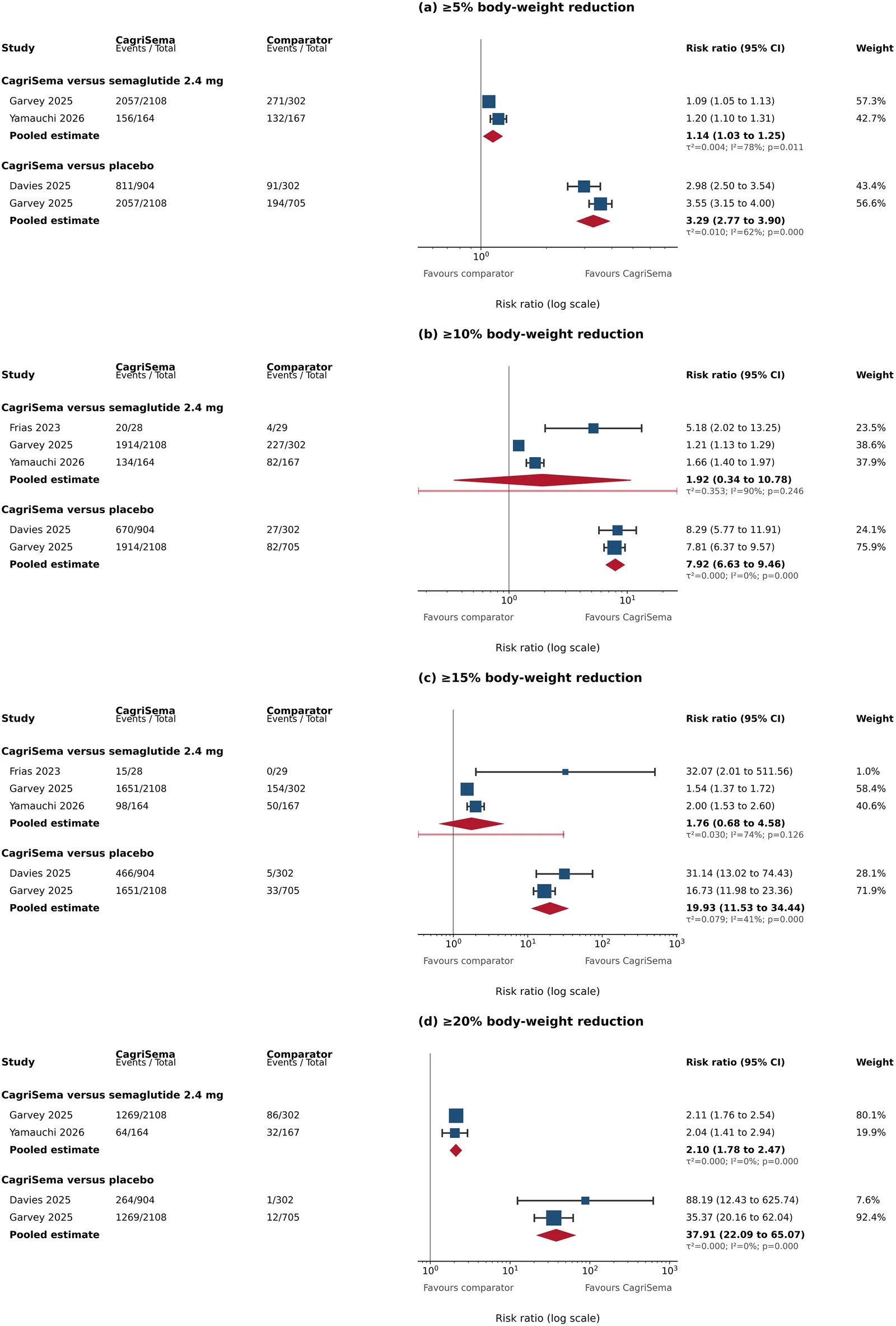
Forest plots of categorical weight-reduction outcomes for CagriSema versus semaglutide 2.4 mg and versus placebo, under the trial-product estimand. **(A)** At least 5% body-weight reduction. **(B)** At least 10%. **(C)** At least 15%. **(D)** At least 20%. Risk ratios are plotted on a logarithmic scale. Responder counts and denominators for every trial, labelled as observed or reconstructed from model-based estimated proportions, are given in **Supplementary Table S6**. Plot conventions as in **Figure 3**. Columns to the left of each plot give the estimated mean change from baseline in each arm with its reported measure of variability, labelled SD or SE, and the analysis denominator; NR denotes that the trial reported no arm-level measure of variability, in which case the standard error used in pooling was derived from the published confidence interval around the treatment difference. Columns to the left of each plot give the number of participants with the event and the analysis denominator in each arm. Counts are observed for Frias 2023 and reconstructed from model-based estimated proportions for the remaining trials, as detailed in **Supplementary Table S6**.

The intermediate thresholds were markedly heterogeneous and are not statistically significant once the small-sample adjustment is applied: 1.92 (95% CI 0.34 to 10.78; I² = 90%) for at least 10% and 1.76 (95% CI 0.68 to 4.58; I² = 74%) for at least 15%. In both cases the heterogeneity is attributable to Frias 2023, a 62-participant phase 2 trial in type 2 diabetes in which the comparator response was very low (4 of 29 and 0 of 29 participants, respectively), producing risk ratios of 5.18 and 32.07 that are incompatible in scale with those from the phase 3a trials. Restricting these outcomes to the phase 3a trials yielded 1.40 (95% CI 1.03 to 1.92) for at least 10% and 1.70 (95% CI 1.32 to 2.19) for at least 15%, both statistically significant and of a magnitude consistent with the other thresholds. The apparent instability of these two outcomes therefore reflects the leverage of one small early-phase trial rather than genuine disagreement among the phase 3a trials.

Versus placebo, the corresponding risk ratios were 3.29 (95% CI 2.77 to 3.90), 7.92 (95% CI 6.63 to 9.46), 19.93 (95% CI 11.53 to 34.44) and 37.91 (95% CI 22.09 to 65.07) for thresholds of at least 5%, 10%, 15% and 20%; the very large estimates at the higher thresholds reflect the near-absent placebo response.

### Waist circumference and blood pressure

CagriSema produced a greater reduction in waist circumference than semaglutide 2.4 mg, at −4.07 cm (95% CI −5.10 to −3.04; I² = 0%), an estimate contributed by the two phase 3a head-to-head trials only; the corresponding HKSJ interval is −6.45 to −1.69 cm (**Figure 5A**).

**Figure 5:**
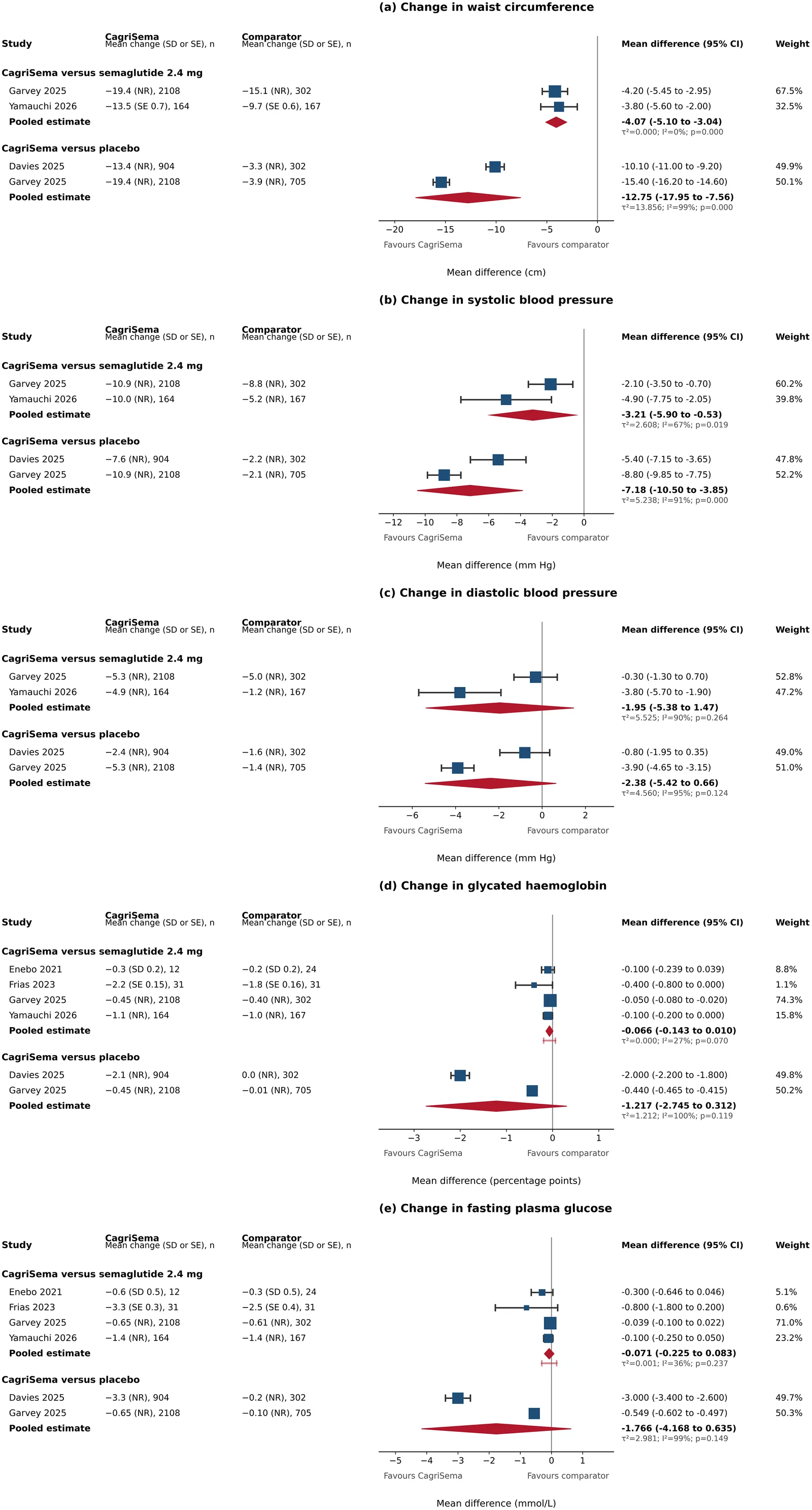
Forest plots of cardiometabolic outcomes for CagriSema versus semaglutide 2.4 mg and versus placebo, under the trial-product estimand. **(A)** Change in waist circumference (cm). **(B)** Change in systolic blood pressure (mm Hg). **(C)** Change in diastolic blood pressure (mm Hg). **(D)** Change in glycated haemoglobin (percentage points). **(E)** Change in fasting plasma glucose (mmol/L). Arm-level inputs are given in **Supplementary Table S5**. Plot conventions as in **Figure 3**. Columns to the left of each plot give the estimated mean change from baseline in each arm with its reported measure of variability, labelled SD or SE, and the analysis denominator; NR denotes that the trial reported no arm-level measure of variability, in which case the standard error used in pooling was derived from the published confidence interval around the treatment difference.

Systolic blood pressure fell further with CagriSema, at −3.21 mm Hg (95% CI −5.90 to −0.53), but the two contributing trials disagreed substantially (τ² = 2.61; I² = 67%; Garvey 2025 −2.1 mm Hg, Yamauchi 2026 −4.9 mm Hg) and the HKSJ interval spans −20.63 to 14.20 mm Hg (**Figure 5B**). For diastolic blood pressure the pooled difference was −1.95 mm Hg (95% CI −5.38 to 1.47), not statistically significant, with severe inconsistency between the two trials (τ² = 5.53; I² = 90%) (**Figure 5C**). Versus placebo, systolic blood pressure fell by −7.18 mm Hg (95% CI −10.50 to −3.85) and diastolic blood pressure by −2.38 mm Hg (95% CI −5.42 to 0.66).

### Glycaemic outcomes

Adding cagrilintide to semaglutide produced no detectable incremental glycaemic benefit. The pooled difference in glycated haemoglobin was −0.07 percentage points (95% CI −0.14 to 0.01; p = 0.07; I² = 27%; prediction interval −0.19 to 0.06) (**Figure 5D**) and in fasting plasma glucose −0.07 mmol/L (95% CI −0.23 to 0.08; I² = 36%) (**Figure 5E**). Both intervals lie well within the prespecified minimal important differences of 0.3 percentage points and 0.5 mmol/L respectively, so these are precise null results rather than inconclusive ones: the data exclude a clinically meaningful incremental glycaemic effect of adding cagrilintide to semaglutide, at least over the durations studied.

Versus placebo, CagriSema reduced glycated haemoglobin by −1.22 percentage points (95% CI −2.75 to 0.31) and fasting plasma glucose by −1.77 mmol/L (95% CI −4.17 to 0.64); both estimates are dominated by the difference in baseline glycaemic status between REDEFINE 1, which excluded diabetes, and REDEFINE 2, which required it (I² = 100% and 99% respectively), and neither should be interpreted as a single transportable effect.

### Patient-reported physical function

Garvey 2025 reports the IWQOL-Lite-CT Physical Function and SF-36v2 Physical Functioning scores only for the comparison of CagriSema with placebo. Garvey 2025 therefore cannot contribute to the CagriSema-versus-semaglutide stratum for these two outcomes, and Yamauchi 2026 is the only trial that provides a head-to-head estimate.

Against semaglutide 2.4 mg, the single available estimate showed no difference in either measure: 1.4 points (95% CI −2.0 to 4.8) for IWQOL-Lite-CT Physical Function and −0.3 points (95% CI −1.2 to 0.6) for SF-36v2 Physical Functioning (**Supplementary Figures S2a**, **S2b**). Both intervals exclude the within-participant meaningful change thresholds used in the trials, so the available head-to-head evidence does not support an incremental benefit of adding cagrilintide on patient-reported physical function, although it rests on one 331-participant trial conducted in an east Asian population.

Against placebo, both measures favoured CagriSema, at 9.41 points (95% CI 2.65 to 16.17) for IWQOL-Lite-CT and 3.15 points (95% CI 1.39 to 4.91) for SF-36v2, with substantial heterogeneity between the two contributing trials (I² = 94% and 88%).

### Safety and tolerability

Safety estimates, together with the event and denominator counts underlying each of them, are shown in **Supplementary Figure S1** and reported trial by trial in **Supplementary Table S7**. All counts below are summed across the trials contributing to each estimate.

Any adverse event was reported in 2,119 of 2,313 participants (91.6%) receiving CagriSema and 457 of 524 (87.2%) receiving semaglutide 2.4 mg (RR 1.03, 95% CI 1.01 to 1.05; I² = 0%), a difference well below the prespecified threshold for an appreciable effect. Gastrointestinal adverse events occurred in 1,792 of 2,313 participants (77.5%) receiving CagriSema and 337 of 524 (64.3%) receiving semaglutide 2.4 mg. The pooled risk ratio was 1.09 (95% CI 0.97 to 1.21; I² = 7%; prediction interval 0.94 to 1.25). The interval crosses the null, so an incremental increase in gastrointestinal burden is neither demonstrated nor excluded. The point estimate is unchanged from the analysis using the DerSimonian–Laird estimator without small-sample adjustment (1.09, 95% CI 1.01 to 1.18), and the difference in statistical significance is attributable entirely to the inferential method rather than to the data. The phase-3a-restricted estimate was 1.07 (95% CI 1.00 to 1.15). Injection-site reactions occurred in 278 of 2,313 participants (12.0%) receiving CagriSema and 19 of 524 (3.6%) receiving semaglutide 2.4 mg (RR 3.14, 95% CI 1.42 to 6.96; I² = 8%). This was the only tolerability signal that remained statistically significant under the primary model and that was consistent in direction across all four contributing trials. Serious adverse events were reported in 212 of 2,301 participants (9.2%) receiving CagriSema and 28 of 500 (5.6%) receiving semaglutide 2.4 mg (RR 0.91, 95% CI 0.08 to 10.80; I² = 71%). Discontinuation due to adverse events occurred in 128 of 2,301 (5.6%) and 16 of 500 (3.2%) respectively (RR 1.35, 95% CI 0.41 to 4.51; I² = 0%). Neoplasms were reported in 145 of 2,270 (6.4%) and 29 of 469 (6.2%) respectively (RR 1.02, 95% CI 0.68 to 1.52; I² = 0%) (**Table 3A**).

**Table 3a:** Summary of pooled effects, CagriSema versus semaglutide 2.4 mg (primary comparison).

Outcome	Trials (k)	Participants	Pooled estimate (95% CI)	p	τ²	I²	95% prediction interval	HKSJ interval
Body weight								
Percentage change in body weight (percentage points)	4	2,835	−6.89 (−8.70 to −5.06)	0.001	0.00	33%	−8.91 to −4.86	−8.70 to −5.06
Absolute change in body weight (kg)	4	2,835	−7.16 (−9.15 to −5.17)	0.001	0.00	38%	−9.27 to −5.05	−9.15 to −5.17
Change in body-mass index (kg/m²)	3	2,773	−2.41 (−2.83 to −1.99)	0.002	0.00	0%	−4.72 to −0.10	−2.83 to −1.99
≥5% body-weight reduction (RR)	2	2,741	1.14 (1.03 to 1.25)	0.011	0.004	78%	—	(0.60 to 2.15)
≥10% body-weight reduction (RR)	3	2,798	1.92 (0.34 to 10.78)	0.246	0.353	90%	uninformative	0.34 to 10.78
≥15% body-weight reduction (RR)	3	2,798	1.76 (0.68 to 4.58)	0.126	0.030	74%	0.10 to 30.19	0.68 to 4.58
≥20% body-weight reduction (RR)	2	2,741	2.10 (1.78 to 2.47)	<0.001	0.00	0%	—	(1.74 to 2.54)
Cardiometabolic								
Change in waist circumference (cm)	2	2,741	−4.07 (−5.10 to −3.04)	<0.001	0.00	0%	—	(−6.45 to −1.69)
Change in systolic blood pressure (mm Hg)	2	2,741	−3.21 (−5.90 to −0.53)	0.019	2.61	67%	—	(−20.63 to 14.20)
Change in diastolic blood pressure (mm Hg)	2	2,741	−1.95 (−5.38 to 1.47)	0.264	5.53	90%	—	(−24.15 to 20.25)
Change in glycated haemoglobin (percentage points)	4	2,835	−0.066 (−0.143 to 0.010)	0.070	0.0004	27%	−0.194 to 0.061	−0.143 to 0.010
Change in fasting plasma glucose (mmol/L)	4	2,835	−0.071 (−0.225 to 0.083)	0.237	0.0014	36%	−0.310 to 0.167	−0.225 to 0.083
Patient-reported physical function								
IWQOL-Lite-CT Physical Function score (points)	1	331	1.40 (−2.00 to 4.80)	0.420	—	—	—	—
SF-36v2 Physical Functioning score (points)	1	331	−0.30 (−1.20 to 0.60)	0.514	—	—	—	—
Safety								
Any adverse event (RR)	4	**2,837**	1.03 (1.01 to 1.05)	0.012	0.00	0%	0.96 to 1.11	1.01 to 1.05
Serious adverse events (RR)	3	**2,801**	0.91 (0.08 to 10.80)	0.888	0.663	71%	uninformative	0.08 to 10.80
Gastrointestinal adverse events (RR)	4	**2,837**	1.09 (0.97 to 1.21)	0.095	0.00	7%	0.94 to 1.25	0.97 to 1.21
Discontinuation due to adverse events (RR)	3	2,801	1.35 (0.41 to 4.51)	0.394	0.031	0%	uninformative	0.41 to 4.51
Injection-site reactions (RR)	4	**2,837**	3.14 (1.42 to 6.96)	0.019	0.111	8%	0.44 to 22.29	1.42 to 6.96
Neoplasms (RR)	2	2,739	1.02 (0.68 to 1.52)	0.934	0.00	0%	—	(0.26 to 3.97)

Bold denotes intervals that exclude the null under the primary model. RR = risk ratio. “Uninformative” denotes a prediction interval spanning values incompatible with any plausible treatment effect. Participant counts are the analysis-set totals of the trials contributing to each estimate and therefore vary by outcome.

These three estimates are inconclusive rather than reassuring. Each rests on few events accrued over a maximum of 68 weeks, and each confidence interval is compatible with both a substantial reduction and a substantial increase in risk: the interval for serious adverse events spans a nine-fold reduction to a ten-fold increase, and the interval for neoplasms spans a one-third reduction to a one-half increase. The available data are therefore insufficient to determine whether the addition of cagrilintide alters the risk of serious adverse events, treatment discontinuation, or neoplasms. They neither demonstrate nor exclude a clinically important difference, and they cannot support any inference of comparable safety. For neoplasms in particular, the maximum follow-up of 68 weeks is far shorter than the latency of most malignancies, so the outcome as measured cannot address the clinical question of malignancy risk.

Against placebo, CagriSema was associated with more adverse events overall (2,758/3,010, 91.6%, versus 838/1,007, 83.2%; RR 1.09, 95% CI 1.03 to 1.16), more gastrointestinal adverse events (2,331/3,010, 77.4%, versus 385/1,007, 38.2%; RR 2.02, 95% CI 1.87 to 2.19), more discontinuations due to adverse events (201/3,010, 6.7%, versus 34/1,007, 3.4%; RR 2.02, 95% CI 1.24 to 3.31) and more injection-site reactions (300/3,010, 10.0%, versus 21/1,007, 2.1%; RR 6.72, 95% CI 1.24 to 36.41). Estimates for serious adverse events (300/3,010, 10.0%, versus 82/1,007, 8.1%; RR 1.14, 95% CI 0.58 to 2.24) and neoplasms (197/3,010, 6.5%, versus 51/1,007, 5.1%; RR 1.28, 95% CI 0.95 to 1.73) are likewise inconclusive (**Table 3B**).

**Table 3b:** Summary of pooled effects, CagriSema versus placebo (contextual comparison).

Outcome	Trials (k)	Participants	Pooled estimate (95% CI)	p	τ²	I²	HKSJ interval
Percentage change in body weight (percentage points)	2	4,019	−16.50 (−24.15 to −8.86)	<0.001	30.26	99%	(−66.06 to 33.05)
Absolute change in body weight (kg)	2	4,019	−17.30 (−25.73 to −8.88)	<0.001	36.80	100%	(−71.94 to 37.33)
Change in body-mass index (kg/m²)	2	4,019	−6.20 (−9.14 to −3.26)	<0.001	4.47	99%	(−25.26 to 12.86)
≥5% body-weight reduction (RR)	2	4,019	3.29 (2.77 to 3.90)	<0.001	0.010	62%	(1.09 to 9.88)
≥10% body-weight reduction (RR)	2	4,019	7.92 (6.63 to 9.46)	<0.001	0.00	0%	(5.71 to 10.98)
≥15% body-weight reduction (RR)	2	4,019	19.93 (11.53 to 34.44)	<0.001	0.080	41%	(0.57 to 692.15)
≥20% body-weight reduction (RR)	2	4,019	37.91 (22.09 to 65.07)	<0.001	0.00	0%	(1.75 to 822.60)
Change in waist circumference (cm)	2	4,019	−12.75 (−17.95 to −7.56)	<0.001	13.86	99%	(−46.43 to 20.92)
Change in systolic blood pressure (mm Hg)	2	4,019	−7.18 (−10.50 to −3.85)	<0.001	5.24	91%	(−28.75 to 14.40)
Change in diastolic blood pressure (mm Hg)	2	4,019	−2.38 (−5.42 to 0.66)	0.124	4.56	95%	(−22.07 to 17.31)
Change in glycated haemoglobin (percentage points)	2	4,019	−1.217 (−2.745 to 0.312)	0.119	1.21	100%	(−11.13 to 8.69)
Change in fasting plasma glucose (mmol/L)	2	4,019	−1.766 (−4.168 to 0.635)	0.149	2.98	99%	(−17.34 to 13.80)
IWQOL-Lite-CT Physical Function score (points)	2	4,019	9.41 (2.65 to 16.17)	0.006	22.48	94%	(−34.42 to 53.24)
SF-36v2 Physical Functioning score (points)	2	4,019	3.15 (1.39 to 4.91)	<0.001	1.42	88%	(−8.27 to 14.57)
Any adverse event (RR)	2	4,017	1.09 (1.03 to 1.16)	0.004	0.001	72%	(0.74 to 1.60)
Serious adverse events (RR)	2	4,017	1.14 (0.58 to 2.24)	0.702	0.208	88%	(0.01 to 90.82)
Gastrointestinal adverse events (RR)	2	4,017	2.02 (1.87 to 2.19)	<0.001	0.00	0%	(1.52 to 2.70)
Discontinuation due to adverse events (RR)	2	4,017	2.02 (1.24 to 3.31)	0.005	0.053	39%	(0.08 to 49.31)
Injection-site reactions (RR)	2	4,017	6.72 (1.24 to 36.41)	0.027	0.943	48%	(0.00 to 384,006)
Neoplasms (RR)	2	4,017	1.28 (0.95 to 1.73)	0.110	0.001	2%	(0.18 to 9.16)

### Sensitivity analyses

Leave-one-out analyses for the primary comparison are shown in **Supplementary Figures S3**, **S4** and tabulated in **Supplementary Table S4**. Percentage and absolute change in body weight, body-mass index, glycated haemoglobin, fasting plasma glucose, discontinuation due to adverse events and neoplasms were stable, with no change in interpretation on removing any single trial. Injection-site reactions lost statistical significance on removal of any single trial other than Enebo 2021, reflecting the small number of events outside the two largest trials. The categorical thresholds of at least 10% and at least 15% were, as described above, dominated by Frias 2023.

Restriction to the three 68-week phase 3a trials is reported for every outcome in **Supplementary Table S4**. Restriction generally reduced heterogeneity and narrowed intervals without reversing the direction of any estimate. Results under the DerSimonian–Laird estimator without the Hartung–Knapp adjustment are also given in **Supplementary Table S4**. Point estimates were essentially unchanged; intervals were narrower, and four outcomes that are not statistically significant under the primary model reach conventional significance under DerSimonian–Laird (glycated haemoglobin, gastrointestinal adverse events, and the thresholds of at least 10% and at least 15%). This comparison quantifies how much of the apparent precision in analyses of this evidence base is an artefact of the estimator rather than a property of the data.

### Trial sequential analysis

Trial sequential analysis for the primary outcome is shown in **Figure 6**. Diversity was 51.9%. Using the externally specified minimal important difference of 3 percentage points and an outcome standard deviation of 9.0 percentage points, the diversity-adjusted required information size was 587 participants, which the accumulated 2,835 randomised participants exceed by a factor of 4.8. The cumulative Z-curve crossed both the conventional significance boundary and the O’Brien–Fleming monitoring boundary after the second trial and remained beyond the monitoring boundary thereafter, reaching −10.5 at the final accumulated information. The required information size is sensitive to the assumed minimal important difference: 1,321 participants for a threshold of 2 percentage points and 211 participants for a threshold of 5 percentage points. The accumulated sample exceeds all three.

**Figure 6:**
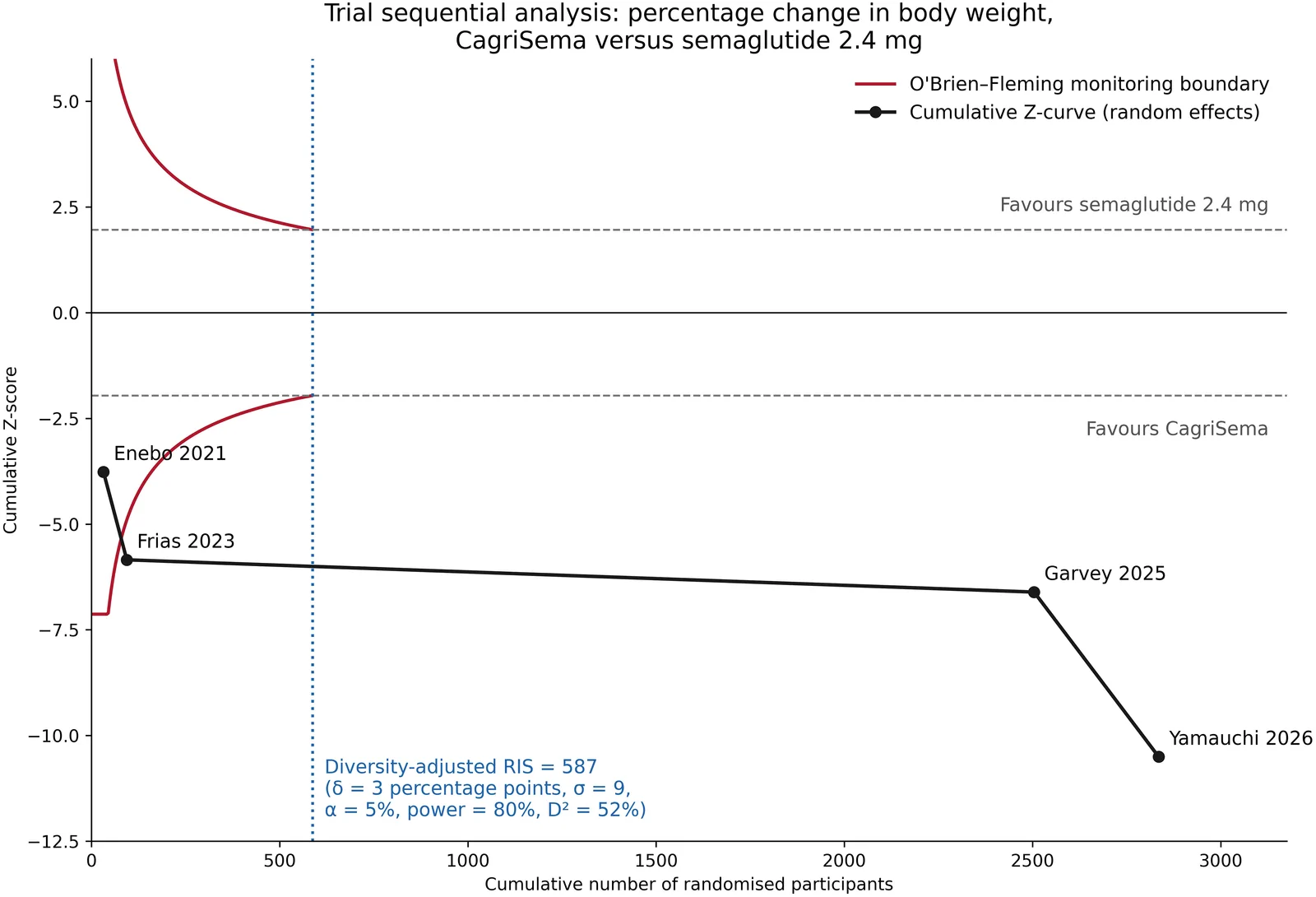
Trial sequential analysis for percentage change in body weight, CagriSema versus semaglutide 2.4 mg. The black line is the cumulative Z-curve from a random-effects cumulative meta-analysis, with trials added in chronological order. The red curve is the O’Brien–Fleming alpha-spending monitoring boundary and the grey dashed lines are the conventional significance boundary (Z = ± 1.96). The blue dotted vertical line marks the diversity-adjusted required information size of 587 participants, calculated from an externally specified minimal important difference of 3 percentage points, an outcome standard deviation of 9.0 percentage points, a two-sided alpha of 5%, 80% power and a diversity of 52%.

These results indicate that the direction of the CagriSema-versus-semaglutide effect on body weight is unlikely to be overturned by further trials. They do not establish the magnitude of the incremental effect, its durability beyond 68 weeks, or its consistency across populations, all of which remain uncertain. The analysis is confined to a single outcome in an evidence base of four trials of differing duration, and is reported as supportive rather than confirmatory.

### Certainty of evidence

The certainty of evidence is summarised in **Table 1a** (CagriSema versus semaglutide 2.4 mg) and **Table 1b** (CagriSema versus placebo), with an outcome-specific explanatory footnote for every downgrade.

For the primary comparison, certainty was high for the proportion of participants achieving at least a 20% body-weight reduction; moderate for percentage and absolute change in body weight, change in body-mass index, waist circumference, glycated haemoglobin, fasting plasma glucose, any adverse event and gastrointestinal adverse events; low for systolic blood pressure, the threshold of at least 5% and injection-site reactions; and very low for diastolic blood pressure, the thresholds of at least 10% and at least 15%, both physical-function outcomes, serious adverse events, discontinuation due to adverse events and neoplasms.

The most frequent reasons for downgrading were indirectness arising from the range of treatment durations contributing to an estimate, inconsistency between the two head-to-head phase 3a trials for the blood-pressure outcomes, and imprecision arising from sparse events. Publication bias was recorded as undetected but not formally assessable for all outcomes; registry searching identified no completed eligible trial without a full publication. Certainty is higher than in the original submission for several body-weight outcomes because harmonising the estimand removed a source of indirectness, and lower for the intermediate weight-reduction thresholds because the small-sample adjustment widened the corresponding intervals.

For the placebo comparison, certainty was high for the thresholds of at least 10% and at least 20% body-weight reduction and for gastrointestinal adverse events, and low or very low for the remaining outcomes, principally because only two trials contribute and because those two trials enrolled populations that differ in diabetes status, producing heterogeneity approaching 100% for most continuous outcomes.

## Discussion

This systematic review and meta-analysis quantifies the incremental effect of adding the long-acting amylin analogue cagrilintide to semaglutide 2.4 mg across the spectrum of adults with overweight or obesity, with and without type 2 diabetes. Three earlier syntheses have examined overlapping evidence. Dutta and colleagues, in 2024, pooled the small phase 1b and phase 2 trials of cagrilintide alone or in combination with semaglutide against placebo or active comparators available through August 2023, and could not include any of the three REDEFINE phase 3a trials that have since defined the field (13). Ahmed and colleagues, in 2026, published a meta-analysis and meta-regression restricted to three randomised trials (n = 3,545) evaluating cagrilintide-based therapies against semaglutide, but did not include the head-to-head REDEFINE 5 trial in an east Asian population and did not undertake a dedicated CagriSema-versus-placebo synthesis, a GRADE assessment, or a trial sequential analysis (14). Gadelmawla and colleagues, also in 2026, reported comparative efficacy and safety of CagriSema against semaglutide monotherapy or placebo across an evidence base closely overlapping our own (15). The contribution of the present review is therefore methodological rather than one of priority. First, we harmonised the estimand across all contributing trials, using the trial-product estimand reported by every included trial, so that pooled estimates answer a single clinical question; this alone reduced heterogeneity for the primary outcome from an I² of 60% under mixed estimands to 33%. Second, we replaced the DerSimonian–Laird estimator with restricted maximum likelihood and a Hartung–Knapp–Sidik–Jonkman small-sample adjustment, and report both models side by side, so that readers can attribute differences between reviews to the estimator rather than to the data. Third, we report the complete prespecified outcome set, including all four weight-reduction thresholds, absolute weight change, body-mass index, diastolic blood pressure and fasting plasma glucose, with an explicit matrix documenting why each trial does or does not contribute to each estimate. Fourth, we provide outcome-specific GRADE justification and a trial sequential analysis whose required information size is anchored on an externally specified minimal important difference rather than on the effect observed in the same synthesis.

The magnitude of additional weight loss attributable to dual incretin and amylin agonism is mechanistically coherent. Semaglutide reduces energy intake primarily through GLP-1 receptors in the hypothalamus and hindbrain, whereas cagrilintide engages amylin and calcitonin receptors in partially distinct brain regions governing both homeostatic and hedonic appetite control (6, 26). The convergence of these complementary pathways provides a plausible biological explanation for the additional appetite suppression observed in the combination groups and is consistent with preclinical mapping work demonstrating non-overlapping neuronal targets for the two molecules (26). That the incremental effect was of similar magnitude whether expressed as a relative change of 6.9 percentage points, an absolute change of 7.2 kg, or a reduction of 2.4 kg/m² in body-mass index, and that the prediction interval excluded values below approximately 5 percentage points, indicates a consistent rather than a marginal biological effect.

Placed in the wider therapeutic context, these findings position CagriSema within the most efficacious tier of contemporary anti-obesity pharmacotherapy. The reference standard against which the combination was compared, semaglutide 2.4 mg, transformed obesity care through the STEP programme and subsequently demonstrated cardiovascular benefit in the SELECT trial, where it reduced major adverse cardiovascular events by approximately one fifth in adults with established cardiovascular disease and overweight or obesity without diabetes (27). The other dominant dual agonist, the GIP and GLP-1 receptor agonist tirzepatide, achieved weight reductions of up to roughly 21% in SURMOUNT-1 (28) and correspondingly robust reductions in adults with type 2 diabetes in SURMOUNT-2 (29). Our synthesis indicates that amylin-based combination therapy delivers weight loss broadly comparable to that seen with tirzepatide in its pivotal trials, including the head-to-head SURMOUNT-5 trial, in which tirzepatide produced a 20.2% weight reduction against 13.7% with semaglutide 2.4 mg at 72 weeks (30). Comparisons with bariatric surgery are not warranted from the current evidence, as no cardiovascular, mortality, long-term weight-maintenance, or body-composition outcomes are yet available for CagriSema. Individuals with type 2 diabetes typically achieve attenuated weight loss relative to those without diabetes, partly owing to concomitant glucose-lowering agents that promote weight gain (31); whether the incremental benefit of adding cagrilintide narrows this gap requires formal subgroup comparison within adequately powered trials or an individual-participant-data synthesis, neither of which was feasible with the currently available published data.

A notable dissociation emerges in the glycaemic domain. Despite clear separation in body weight, no incremental effect on glycated haemoglobin or fasting plasma glucose was detectable, and both confidence intervals lay entirely within the prespecified minimal important differences of 0.3 percentage points and 0.5 mmol/L. These are therefore precise null results rather than inconclusive ones, and they exclude a clinically meaningful incremental glycaemic effect over the durations studied. This pattern is biologically plausible: semaglutide exerts potent direct glucose-lowering effects through glucose-dependent insulinotropic and glucagonostatic actions, whereas the glycaemic contribution of cagrilintide is mediated more indirectly through weight loss, delayed gastric emptying, and postprandial glucagon suppression (32, 33). In populations already deriving strong glycaemic benefit from semaglutide, the incremental glycaemic gain from added amylin agonism is therefore modest, even where weight loss diverges substantially. The dissociation reinforces the principle that weight loss and glycaemic control, although related, are not interchangeable endpoints in obesity–diabetes therapeutics (34). A comparable dissociation was seen for patient-reported physical function: the only trial reporting a head-to-head comparison found no difference on either the IWQOL-Lite-CT or the SF-36v2, with intervals excluding the within-participant thresholds regarded as meaningful in those trials.

The greater reductions in waist circumference and systolic blood pressure observed with combination therapy represent improvements in surrogate cardiometabolic outcomes rather than in hard clinical endpoints. Whether these translate into reductions in major adverse cardiovascular events, mortality, or long-term weight maintenance remains unproven for CagriSema and represents a critical evidence gap, given that such outcome data already exist for semaglutide monotherapy (27). The tolerability trade-off is more nuanced than the point estimates alone suggest. Injection-site reactions were consistently and substantially more frequent with combination therapy and were the only tolerability signal that remained statistically significant under the primary model. Gastrointestinal adverse events, by contrast, were numerically more frequent (77.5% versus 64.3%) but the pooled interval crossed the null once the small-sample adjustment was applied, so the evidence neither demonstrates nor excludes an incremental gastrointestinal burden, a pattern consistent with class effects of incretin-based and amylin-based agents and with the flexible, individualized dose-titration approach adopted in the included trials (35). An additional consideration that warrants vigilance is body composition: as with all highly effective incretin-based therapies, a proportion of weight lost is lean mass, raising questions about muscle health that future trials of CagriSema should address directly through standardised body-composition and functional assessment (36).

Our findings should be interpreted alongside the recent large network meta-analysis by Nong and colleagues, which synthesised 262 randomised trials involving 99,791 participants across 19 anti-obesity drugs (37). In that network, CagriSema and tirzepatide produced the largest one-year weight reductions relative to lifestyle modification alone (−14.8% and −14.9%, respectively), placing the combination at the upper end of currently available pharmacological options, a finding consistent in direction and magnitude with our head-to-head estimate against semaglutide 2.4 mg. Our review complements that indirect comparison by providing a direct, pairwise synthesis restricted to head-to-head randomised evidence, thereby avoiding the transitivity assumptions inherent to network meta-analysis. The Nong network also identified CagriSema among the agents with the highest risk of treatment discontinuation due to adverse events, mirroring the tolerability direction in our synthesis, although in our head-to-head analysis the discontinuation estimate was imprecise and not statistically significant. Taken together, the convergence between our direct pairwise estimates and the broader indirect network suggests that the incremental benefit of adding cagrilintide to semaglutide is genuine and clinically meaningful for body weight, while its magnitude for cardiometabolic and patient-reported endpoints will remain uncertain until further adequately powered head-to-head trials report.

## Clinical implications

Several implications follow for clinical practice and research. Because none of the included trials enrolled semaglutide non-responders or evaluated cagrilintide added to established semaglutide therapy, the present evidence supports combination initiation of cagrilintide with semaglutide as a randomised treatment strategy from the outset, rather than stepwise intensification after inadequate response to semaglutide monotherapy. Whether a sequential strategy of adding cagrilintide after an insufficient response to semaglutide would deliver similar incremental benefit remains untested and should be evaluated in dedicated trials. The inclusion of trials enrolling adults with and without type 2 diabetes, and of trials conducted in both predominantly White global populations and an east Asian population, broadens the applicability of the findings, although the small number of eligible trials precluded formal quantitative testing of whether the incremental effect differs across these subgroups; ethnicity-specific obesity definitions and risk thresholds should be considered when interpreting effect sizes for individual populations.

These findings can be placed against contemporary obesity-management recommendations in three ways. First, current guidance frames pharmacotherapy around categorical weight-loss thresholds rather than mean change, because obesity-related complications improve progressively as greater degrees of weight loss are achieved (38). Our threshold analyses speak directly to this: roughly twice as many patients reached a 20% reduction with CagriSema as with semaglutide 2.4 mg, the degree of weight loss at which multiple complications of obesity begin to resolve. Second, guidance increasingly recommends selecting an agent according to the dominant comorbidity (34). Our finding of no incremental glycaemic benefit, with intervals lying entirely within the minimal important difference, argues against preferring CagriSema over semaglutide where glycaemic control rather than weight is the principal treatment target, and is consistent with the observation that the incremental effect of cagrilintide is mediated largely through weight rather than through direct glucose-lowering action. Third, current recommendations for semaglutide 2.4 mg rest in part on demonstrated cardiovascular outcome benefit (27), for which no equivalent evidence yet exists for CagriSema. The surrogate improvements in blood pressure and waist circumference observed here cannot substitute for outcome data. On present evidence CagriSema is best regarded as an option for patients in whom the treatment goal is the magnitude of weight loss itself, and in whom the additional injection-site and gastrointestinal burden is acceptable, with its position in the treatment framework remaining provisional until cardiovascular outcome and body-composition data are available.

## Limitations

This analysis has limitations that temper the strength of inference. First, only five randomised controlled trials met the eligibility criteria, and several outcomes are informed by only two. Where only two trials contribute, the between-study variance is estimated with a single degree of freedom, the Hartung–Knapp interval becomes uninterpretably wide, and we therefore report the restricted maximum likelihood estimate with a Wald-type interval as the primary result while using the Hartung–Knapp interval to judge imprecision under GRADE. Estimates for waist circumference, blood pressure, the 5% and 20% thresholds and neoplasms should be read with that constraint in mind, and the entire CagriSema-versus-placebo stratum, in which every outcome rests on two trials enrolling populations that differ in diabetes status, is best regarded as a descriptive benchmark rather than a set of precise effect estimates.

Second, the included trials differed markedly in phase, treatment duration, geographic setting, and baseline glycaemic status. Because treatment effects on body weight and cardiometabolic outcomes accrue progressively, pooling end-of-treatment estimates from trials of 20, 32 and 68 weeks conflates the magnitude of the treatment effect with its temporal maturity. We quantified this by restricting every outcome to the three 68-week phase 3a trials; restriction generally reduced heterogeneity and narrowed intervals without reversing the direction of any estimate, but it cannot eliminate the underlying limitation. The same applies to the safety analyses, in which risk ratios are pooled across substantially different exposure periods and are therefore not equivalent to incidence-rate comparisons.

Third, harmonising the estimand removed a source of heterogeneity but introduced a constraint of its own. The trial-product estimand estimates the effect of treatment taken as intended and is therefore less conservative than the treatment-policy estimand, which reflects the effect of assignment regardless of discontinuation. Our primary estimates consequently describe the effect achievable under sustained treatment rather than under routine prescribing. The treatment-policy sensitivity analysis, feasible only for the primary outcome and only for three trials, yielded a smaller incremental effect of 5.58 percentage points, and clinicians should regard the true incremental benefit in practice as lying between the two.

Fourth, the categorical weight-reduction thresholds required reconstruction of event numbers from model-based estimated proportions for all trials except Frias 2023, because the source publications report estimated rather than observed proportions. This is a recognised approximation that slightly understates uncertainty. In addition, the estimates for the 10% and 15% thresholds were dominated by a single 62-participant phase 2 trial in which the comparator response was very low, and neither reached statistical significance under the primary model despite both being significant when restricted to the phase 3a trials.

Fifth, because Garvey 2025 (REDEFINE 1) was a four-arm trial contributing its CagriSema arm to both the semaglutide and placebo strata, the two comparator-stratified pooled estimates are not statistically independent, and formal subgroup interaction testing between them was not performed. Several secondary estimates were sensitive to the omission of individual large trials in leave-one-out analysis, indicating that they are driven disproportionately by single studies. Although the overall risk of bias was low for the three largest contributing trials, Enebo 2021 raised some concerns for selection of the reported result owing to its exploratory phase 1b design, and Yamauchi 2026 raised some concerns for missing outcome data given differential discontinuation in a smaller sample.

Sixth, patient-reported physical function against semaglutide 2.4 mg rests on a single 331-participant trial conducted in Japan and Taiwan, because Garvey 2025 measured both instruments but reports them only against placebo. The absence of a demonstrated incremental benefit on these outcomes should therefore be read as an absence of adequate head-to-head evidence rather than as established equivalence.

Seventh, follow-up was insufficient to evaluate weight-loss maintenance during continued therapy or to characterise weight regain after discontinuation. The longest included trials extended to 68 weeks of active treatment with a post-treatment observation window of a few weeks, whereas substantial weight regain and reversal of cardiometabolic gains after cessation of incretin-based therapy typically manifest over the following year (39). The durability of CagriSema-mediated weight loss beyond one year, and the trajectory of body weight, cardiometabolic risk factors and lean mass after treatment stops, cannot be inferred from the current evidence base.

Finally, although all five trials were sponsored by a single manufacturer and formal statistical assessment of publication bias is uninterpretable with fewer than ten studies, registry searching identified no completed eligible trial lacking a full publication. The absence of a statistically significant difference in serious adverse events or neoplasms should not be interpreted as evidence of equivalence: the confidence intervals are wide, events are sparse, and follow-up of at most 68 weeks is far shorter than the latency of most malignancies. The absence of long-term cardiovascular and mortality outcomes, together with limited body-composition data, further constrains conclusions about the full clinical value of combination therapy.

## Conclusion

In this meta-analysis comparing CagriSema with semaglutide 2.4 mg monotherapy under a harmonised trial-product estimand, the addition of cagrilintide produced clinically meaningful additional weight loss, whether expressed as relative change, absolute change or body-mass index, and roughly doubled the proportion of patients reaching a 20% reduction. Modest incremental reductions in waist circumference and systolic blood pressure were also observed. No incremental benefit was demonstrated for glycated haemoglobin, fasting plasma glucose, or patient-reported physical function; for the glycaemic outcomes the intervals were narrow enough to exclude a clinically meaningful effect, whereas for physical function the evidence rests on a single trial. These gains were accompanied by consistently more frequent injection-site reactions and, at most, a modest increase in gastrointestinal adverse events. For serious adverse events and neoplasms the evidence is inconclusive: events were few, follow-up extended to at most 68 weeks, and the confidence intervals are compatible with both a substantial reduction and a substantial increase in risk. No conclusion about the comparative safety of the two regimens for these outcomes can be drawn from the present data. Larger and longer head-to-head trials with cardiovascular outcome and body-composition endpoints are needed to define the durable clinical role of cagrilintide–semaglutide combination therapy.

## Data Availability

The original contributions presented in the study are included in the article/**Supplementary Material**. Further inquiries can be directed to the corresponding author.
