# Supplementary material for: CagriSema versus Semaglutide 2.4 mg monotherapy for weight management in adults with overweight or obesity, with or without type 2 diabetes: a systematic review, meta-analysis, and trial sequential analysis
Source: Front Endocrinol (Lausanne). 2026 Oct 2;17:1903335. doi: 10.3389/fendo.2026.1903335 (PMC13673521; doi:10.3389/fendo.2026.1903335)
Supplement: Supplementary file 1 [file SupplementaryFile1.docx]

**Supplementary Appendix**

**Supplementary Table S1:** Search string used for databases

**Supplementary Table S2.** PRISMA checklist

**Supplementary Table S3.** Outcome availability by trial

CagriSema versus semaglutide 2.4 mg

**Supplementary Table S4.** Sensitivity analyses (CagriSema versus semaglutide 2.4 mg)

**Supplementary Table S5.** Arm-level data underlying the continuous outcomes. For each trial and each arm, the estimated mean change from baseline with its reported measure of variability and the analysis denominator, the treatment difference as published, and the standard error used in pooling. Values are under the trial-product estimand. NR = no arm-level measure of variability reported; in these cases the standard error used in pooling was derived from the published confidence interval around the treatment difference. "Derived" denotes a treatment difference computed by the review authors from arm-level means and standard deviations. Source column refers to the table of the original publication from which values were extracted.

**Supplementary Table S6.** Responder counts underlying the categorical weight-reduction outcomes. For each trial and each arm, the number of responders and the analysis denominator, labelled as observed where the trial reported raw counts and as reconstructed where counts were derived as the model-based estimated proportion multiplied by the analysis-set denominator. A 0.5 continuity correction was applied to the Frias 2023 ≥15% comparison, in which no comparator participant reached the threshold.

**Supplementary Table S7.** Event counts underlying every safety estimate. For each trial and each arm, the number of participants with at least one event and the safety analysis-set denominator, with the trial-level risk ratio. Outcomes with no events in either arm are shown as not estimable and did not contribute to the corresponding pooled estimate.

**Supplementary Figure S1.** Forest plots of safety outcomes for CagriSema versus semaglutide 2.4 mg and versus placebo. (a) Any adverse event. (b) Serious adverse events. (c) Gastrointestinal adverse events. (d) Discontinuation due to adverse events. (e) Injection-site reactions. (f) Neoplasms. Risk ratios are plotted on a logarithmic scale; a risk ratio below 1 favours CagriSema. Event counts and denominators for every trial are given in Supplementary Table S7. Plot conventions as in Figure 3.

**Supplementary Figure S2.** Forest plots of patient-reported physical function for CagriSema versus semaglutide 2.4 mg and versus placebo. (a) Change in IWQOL-Lite-CT Physical Function score. (b) Change in SF-36v2 Physical Functioning score. Garvey 2025 reports both instruments only against placebo and therefore contributes to the placebo stratum only. Arm-level inputs are given in Supplementary Table S5. Plot conventions as in Figure 3.

**Supplementary Figure S3.** Leave-one-out sensitivity analyses for efficacy outcomes, CagriSema versus semaglutide 2.4 mg, for every outcome informed by three or more trials. (a) Percentage change in body weight. (b) Absolute change in body weight. (c) Change in body-mass index. (d) Change in glycated haemoglobin. (e) Change in fasting plasma glucose. (f) At least 10% body-weight reduction. (g) At least 15% body-weight reduction. Each row shows the pooled estimate with the named trial omitted; the diamond and shaded band show the estimate and confidence interval with all trials included.

**Supplementary Figure S4.** Leave-one-out sensitivity analyses for safety outcomes, CagriSema versus semaglutide 2.4 mg. (a) Any adverse event. (b) Serious adverse events. (c) Gastrointestinal adverse events. (d) Discontinuation due to adverse events. (e) Injection-site reactions. Plot conventions as in Supplementary Figure S3.

**Supplementary Table S1:** Search string used for databases

| **Database / Registry** | **Search String** | **Search Field** | **Records Retrieved** |
| --- | --- | --- | --- |
| **PubMed** | ("CagriSema"[All Fields] OR "cagrilintide"[All Fields] OR "cagrilintide"[Supplementary Concept] OR "NNC0174-0833"[All Fields] OR "NNC0174 0833"[All Fields] OR "AM833"[All Fields] OR (("amylin"[All Fields] OR "amylin analog*"[All Fields] OR "amylin analogue*"[All Fields]) AND ("semaglutide"[All Fields] OR "glucagon-like peptide*"[All Fields] OR "GLP-1"[All Fields]))) | All Fields | 162 |
| **Embase** | ('cagrisema':ti,ab,kw OR 'cagrilintide'/exp OR 'cagrilintide':ti,ab,kw OR 'nnc0174-0833':ti,ab,kw OR 'am833':ti,ab,kw OR (('amylin analog*':ti,ab,kw OR 'amylin analogue*':ti,ab,kw) AND ('semaglutide'/exp OR 'glucagon-like peptide*':ti,ab,kw OR 'glp-1':ti,ab,kw))) | Title, Abstract, Keywords | 440 |
| **Cochrane CENTRAL** | (CagriSema OR cagrilintide OR "NNC0174-0833" OR "NNC0174 0833" OR AM833 OR (("amylin analog*" OR "amylin analogue*") AND (semaglutide OR "GLP-1" OR "glucagon-like peptide*"))) | Title, Abstract, Keyword | 96 |
| **ScienceDirect** | (CagriSema OR cagrilintide OR "NNC0174-0833" OR AM833) OR (("amylin analog" OR "amylin analogue") AND (semaglutide OR "GLP-1" OR "glucagon-like peptide")) | Title, Abstract, Keywords | 853 |
| **ClinicalTrials.gov** | CagriSema OR cagrilintide OR "NNC0174-0833" OR "NNC0174 0833" OR AM833 OR ("amylin analogue" OR "amylin analog" OR "amylin analogues" OR "amylin analogs") AND (semaglutide OR "GLP-1" OR "glucagon-like peptide" OR "glucagon-like peptides") | Other terms (free-text) | 131 |
| **WHO ICTRP** | CagriSema OR cagrilintide OR "NNC0174-0833" OR AM833 OR ("amylin analogue" AND semaglutide) OR ("amylin analog" AND semaglutide) OR ("amylin analogue" AND "GLP-1") OR ("amylin analog" AND "GLP-1") | Intervention | 78 |
| **Total** |  |  | **1,760** |

All searches were run on 30 June 2026.

**Supplementary Table S2.** PRISMA checklist

| **Section / Component** | **Page Number(s)** |
| --- | --- |
| **Title** | 1 |
| **Other Information* (Acknowledgments, Funding, Competing Interests, Ethics Approval, Consent for Publication, Data Availability, Author Contributions)** | 2–3 |
| **Abstract** | 4 |
| **Introduction** | 5–6 |
| **Methods** | 6–11 |
| **— Protocol and registration** | 6 |
| **— Eligibility criteria** | 6–7 |
| **— Information sources and search strategy** | 7 |
| **— Study selection** | 7 |
| **— Data extraction and risk of bias assessment** | 7–8 |
| **— Outcomes and effect measures** | 8–9 |
| **— Estimands** | 9 |
| **— Statistical analysis** | 9–10 |
| **— Trial sequential analysis** | 10–11 |
| **— Certainty of evidence** | 11 |
| **Results** | 12–18 |
| **— Study selection** | 12 |
| **— Characteristics of included studies** | 12 |
| **— Risk of bias** | 12–13 |
| **— Primary outcome: percentage change in body weight** | 13 |
| **— Absolute body weight and body-mass index** | 13 |
| **— Categorical weight-reduction responders** | 14 |
| **— Waist circumference and blood pressure** | 14 |
| **— Glycaemic outcomes** | 14–15 |
| **— Patient-reported physical function** | 15 |
| **— Safety and tolerability** | 15–16 |
| **— Sensitivity analyses** | 16–17 |
| **— Trial sequential analysis** | 17 |
| **— Certainty of evidence** | 17–18 |
| **Discussion** | 18–24 |
| **— Clinical Implications** | 21–22 |
| **— Limitations** | 22–23 |
| **— Conclusion** | 23–24 |
| **References** | 24–28 |

**Supplementary Table S3.** Outcome availability by trial

CagriSema versus semaglutide 2.4 mg

| **Outcome** | **Enebo 2021** | **Frias 2023** | **Garvey 2025** | **Yamauchi 2026** |
| --- | --- | --- | --- | --- |
| **Percentage change in body weight** | R | R | R | R |
| **Absolute change in body weight (kg)** | R | R | R | R |
| **Change in body-mass index** | R ᵃ | NR | R | R |
| **≥5% body-weight reduction** | NR | NR | R | R |
| **≥10% body-weight reduction** | NR | R | R | R |
| **≥15% body-weight reduction** | NR | R | R | R |
| **≥20% body-weight reduction** | NR | NR | R | R |
| **Waist circumference** | NM | NM | R | R |
| **Systolic blood pressure** | NE ᵇ | NE ᶜ | R | R |
| **Diastolic blood pressure** | NE ᵇ | NE ᵇ | R | R |
| **Glycated haemoglobin** | R ᵃ | R | R | R |
| **Fasting plasma glucose** | R ᵃ | R | R ᵈ | R |
| **IWQOL-Lite-CT Physical Function** | NM | NM | NR ᵉ | R |
| **SF-36v2 Physical Functioning** | NM | NM | NR ᵉ | R |
| **Any adverse event** | R | R | R | R |
| **Serious adverse events** | NE ᶠ | R | R | R |
| **Gastrointestinal adverse events** | R | R | R | R |
| **Discontinuation due to adverse events** | NE ᶠ | R | R | R |
| **Injection-site reactions** | R | R | R | R |
| **Neoplasms** | NR | NE ᶠ | R | R |

R = reported and used; NR = measured or measurable but not reported; NM = not measured; NE = reported but not estimable (no variance estimate, or no events in either arm).

ᵃ Derived from arm-level means and standard deviations reported in Table 3 of Enebo 2021. The CagriSema arm is cohort 5 (cagrilintide 2.4 mg plus semaglutide 2.4 mg, n = 12); the comparator is pooled placebo plus semaglutide 2.4 mg (cohorts 1–5, n = 20, for body weight and body-mass index; cohorts 1–6, n = 24, for glycated haemoglobin and fasting plasma glucose, because the cohort 1–5 pool is not reported for those outcomes).

ᵇ Reported graphically in the supplementary appendix without a numerical estimate of variability, so a mean difference and its variance cannot be derived.

ᶜ Arm-level mean changes are reported in the main text (−13.0 mm Hg with CagriSema versus +1.0 mm Hg with semaglutide) but without any measure of variability, so the comparison cannot be pooled.

ᵈ Reported in mg/dL and converted to mmol/L for pooling.

ᵉ Measured in the trial and reported for the comparison with placebo only; the semaglutide and cagrilintide columns of Tables S4 and S5 of the supplementary appendix are not populated. **This is the correction described in the Results.**

ᶠ No events in either arm, so the risk ratio is not estimable and the trial is excluded from the pooled estimate for that outcome.

**CagriSema versus placebo**

All twenty outcomes are reported by both Davies 2025 and Garvey 2025 and contribute to every pooled estimate in this stratum.

**Supplementary Table S4.** Sensitivity analyses (CagriSema versus semaglutide 2.4 mg)

| **Outcome** | **Primary (REML + HKSJ)** | **DerSimonian–Laird, no small-sample adjustment** | **Phase 3a trials only** |
| --- | --- | --- | --- |
| **Percentage change in body weight** | −6.89 (−8.70 to −5.06) | −7.11 (−8.44 to −5.79) | −6.57 (−7.56 to −5.59) |
| **Absolute change in body weight (kg)** | −7.16 (−9.15 to −5.17) | −7.30 (−8.73 to −5.88) | −6.89 (−7.92 to −5.85) |
| **Change in body-mass index** | −2.41 (−2.83 to −1.99) | −2.41 (−2.77 to −2.05) | −2.40 (−2.77 to −2.03) |
| **≥5% body-weight reduction** | 1.14 (1.03 to 1.25) | 1.14 (1.03 to 1.25) | 1.14 (1.03 to 1.25) |
| **≥10% body-weight reduction** | 1.92 (0.34 to 10.78) | 1.64 (1.13 to 2.38) | 1.40 (1.03 to 1.92) |
| **≥15% body-weight reduction** | 1.76 (0.68 to 4.58) | 1.81 (1.26 to 2.61) | 1.70 (1.32 to 2.19) |
| **≥20% body-weight reduction** | 2.10 (1.78 to 2.47) | 2.10 (1.78 to 2.47) | 2.10 (1.78 to 2.47) |
| **Change in waist circumference** | −4.07 (−5.10 to −3.04) | −4.07 (−5.10 to −3.04) | −4.07 (−5.10 to −3.04) |
| **Change in systolic blood pressure** | −3.21 (−5.90 to −0.53) | −3.21 (−5.90 to −0.53) | −3.21 (−5.90 to −0.53) |
| **Change in diastolic blood pressure** | −1.95 (−5.38 to 1.47) | −1.95 (−5.38 to 1.47) | −1.95 (−5.38 to 1.47) |
| **Change in glycated haemoglobin** | −0.066 (−0.143 to 0.010) | −0.074 (−0.129 to −0.019) | −0.054 (−0.083 to −0.025) |
| **Change in fasting plasma glucose** | −0.071 (−0.225 to 0.083) | −0.091 (−0.204 to 0.021) | −0.048 (−0.104 to 0.009) |
| **Any adverse event** | 1.03 (1.01 to 1.05) | 1.03 (1.00 to 1.07) | 1.03 (0.99 to 1.07) |
| **Serious adverse events** | 0.91 (0.08 to 10.80) | 0.91 (0.28 to 2.96) | 1.12 (0.33 to 3.85) |
| **Gastrointestinal adverse events** | 1.09 (0.97 to 1.21) | 1.09 (1.01 to 1.18) | 1.07 (1.00 to 1.15) |
| **Discontinuation due to adverse events** | 1.35 (0.41 to 4.51) | 1.40 (0.81 to 2.43) | 1.46 (0.84 to 2.56) |
| **Injection-site reactions** | 3.14 (1.42 to 6.96) | 3.19 (1.92 to 5.29) | 4.35 (2.43 to 7.80) |
| **Neoplasms** | 1.02 (0.68 to 1.52) | 1.02 (0.68 to 1.52) | 1.02 (0.68 to 1.52) |

Bold marks estimates whose statistical significance differs from that of the primary analysis. The DerSimonian–Laird column reproduces the estimator used in the original submission and in previously published syntheses of this evidence base, and is provided so that readers can attribute differences between reviews to the estimator rather than to the data.

Leave-one-out results for every outcome with three or more contributing trials are shown in Supplementary Figure S3.

**Supplementary Table S5.** Arm-level data underlying the continuous outcomes. For each trial and each arm, the estimated mean change from baseline with its reported measure of variability and the analysis denominator, the treatment difference as published, and the standard error used in pooling. Values are under the trial-product estimand. NR = no arm-level measure of variability reported; in these cases the standard error used in pooling was derived from the published confidence interval around the treatment difference. "Derived" denotes a treatment difference computed by the review authors from arm-level means and standard deviations. Source column refers to the table of the original publication from which values were extracted.

| **Outcome** | **Trial** | **Comparison** | **CagriSema: mean change (variability), n** | **Comparator: mean change (variability), n** | **Treatment difference (95% CI)** | **SE used in pooling** | **Source** |
| --- | --- | --- | --- | --- | --- | --- | --- |
| **Percentage change in body weight (%)** | Enebo 2021 | CagriSema vs semaglutide 2.4 mg | −17.1 (SE 1.5), n=12 | −9.8 (SE 1.2), n=20 | −7.4 (−11.2 to −3.5) | 1.9643 | Table 3 |
|  | Frias 2023 | CagriSema vs semaglutide 2.4 mg | −15.6 (SE 1.26), n=31 | −5.1 (SE 1.26), n=31 | −10.5 (−14.1 to −7) | 1.8113 | Table 2 |
|  | Garvey 2025 | CagriSema vs semaglutide 2.4 mg | −22.7 (NR), n=2108 | −16.1 (NR), n=302 | −6.6 (−7.7 to −5.4) | 0.5867 | Table S5 |
|  | Yamauchi 2026 | CagriSema vs semaglutide 2.4 mg | −18.4 (SE 0.7), n=164 | −11.9 (SE 0.7), n=167 | −6.5 (−8.4 to −4.6) | 0.9694 | Table 2 |
|  | Davies 2025 | CagriSema vs placebo | −15.7 (NR), n=904 | −3.1 (NR), n=302 | −12.6 (−13.4 to −11.7) | 0.4337 | Table S4 |
|  | Garvey 2025 | CagriSema vs placebo | −22.7 (NR), n=2108 | −2.3 (NR), n=705 | −20.4 (−21.1 to −19.7) | 0.3571 | Table S5 |
| **Absolute change in body weight (kg)** | Enebo 2021 | CagriSema vs semaglutide 2.4 mg | −15.9 (SE 1.4), n=12 | −8.9 (SE 1.1), n=20 | −7 (−10.6 to −3.4) | 1.8368 | Table 3 |
|  | Frias 2023 | CagriSema vs semaglutide 2.4 mg | −16.3 (SE 1.3), n=31 | −5.3 (SE 1.3), n=31 | −10.9 (−14.7 to −7.2) | 1.9133 | Table 2 |
|  | Garvey 2025 | CagriSema vs semaglutide 2.4 mg | −24.1 (NR), n=2108 | −16.9 (NR), n=302 | −7.2 (−8.4 to −5.9) | 0.6378 | Table S5 |
|  | Yamauchi 2026 | CagriSema vs semaglutide 2.4 mg | −17.2 (NR), n=164 | −11.0 (NR), n=167 | −6.2 (−8.1 to −4.4) | 0.9439 | Table 2 |
|  | Davies 2025 | CagriSema vs placebo | −16.2 (NR), n=904 | −3.2 (NR), n=302 | −13 (−13.9 to −12.1) | 0.4592 | Table S4 |
|  | Garvey 2025 | CagriSema vs placebo | −24.1 (NR), n=2108 | −2.5 (NR), n=705 | −21.6 (−22.3 to −20.8) | 0.3827 | Table S5 |
| **Change in BMI (kg/m²)** | Enebo 2021 | CagriSema vs semaglutide 2.4 mg | −5.6 (SD 1.7), n=12 | −3.1 (SD 2.1), n=20 | −2.50 (derived) | 0.6792 | Table 3 |
|  | Garvey 2025 | CagriSema vs semaglutide 2.4 mg | −8.6 (NR), n=2108 | −6.1 (NR), n=302 | −2.5 (−3 to −2.1) | 0.2296 | Table S5 |
|  | Yamauchi 2026 | CagriSema vs semaglutide 2.4 mg | −6.2 (NR), n=164 | −4.0 (NR), n=167 | −2.2 (−2.9 to −1.6) | 0.3316 | Table 2 |
|  | Davies 2025 | CagriSema vs placebo | −5.8 (NR), n=904 | −1.1 (NR), n=302 | −4.7 (−5 to −4.3) | 0.1786 | Table S4 |
|  | Garvey 2025 | CagriSema vs placebo | −8.6 (NR), n=2108 | −0.9 (NR), n=705 | −7.7 (−8 to −7.4) | 0.1531 | Table S5 |
| **Change in waist circumference (cm)** | Garvey 2025 | CagriSema vs semaglutide 2.4 mg | −19.4 (NR), n=2108 | −15.1 (NR), n=302 | −4.2 (−5.5 to −3) | 0.6378 | Table S5 |
|  | Yamauchi 2026 | CagriSema vs semaglutide 2.4 mg | −13.5 (SE 0.7), n=164 | −9.7 (SE 0.6), n=167 | −3.8 (−5.6 to −2) | 0.9184 | Table 2 |
|  | Davies 2025 | CagriSema vs placebo | −13.4 (NR), n=904 | −3.3 (NR), n=302 | −10.1 (−11 to −9.2) | 0.4592 | Table S4 |
|  | Garvey 2025 | CagriSema vs placebo | −19.4 (NR), n=2108 | −3.9 (NR), n=705 | −15.4 (−16.2 to −14.6) | 0.4082 | Table S5 |
| **Change in systolic BP (mm Hg)** | Garvey 2025 | CagriSema vs semaglutide 2.4 mg | −10.9 (NR), n=2108 | −8.8 (NR), n=302 | −2.1 (−3.5 to −0.7) | 0.7143 | Table S5 |
|  | Yamauchi 2026 | CagriSema vs semaglutide 2.4 mg | −10.0 (NR), n=164 | −5.2 (NR), n=167 | −4.9 (−7.7 to −2) | 1.4541 | Table 2 |
|  | Davies 2025 | CagriSema vs placebo | −7.6 (NR), n=904 | −2.2 (NR), n=302 | −5.4 (−7.2 to −3.7) | 0.8929 | Table S4 |
|  | Garvey 2025 | CagriSema vs placebo | −10.9 (NR), n=2108 | −2.1 (NR), n=705 | −8.8 (−9.9 to −7.8) | 0.5357 | Table S5 |
| **Change in diastolic BP (mm Hg)** | Garvey 2025 | CagriSema vs semaglutide 2.4 mg | −5.3 (NR), n=2108 | −5.0 (NR), n=302 | −0.3 (−1.3 to 0.7) | 0.5102 | Table S5 |
|  | Yamauchi 2026 | CagriSema vs sem |  |  |  |  |  |

**Supplementary Table S6.** Responder counts underlying the categorical weight-reduction outcomes. For each trial and each arm, the number of responders and the analysis denominator, labelled as observed where the trial reported raw counts and as reconstructed where counts were derived as the model-based estimated proportion multiplied by the analysis-set denominator. A 0.5 continuity correction was applied to the Frias 2023 ≥15% comparison, in which no comparator participant reached the threshold.

| **Threshold** | **Trial** | **Comparison** | **CagriSema n/N (%)** | **Comparator n/N (%)** | **Type** | **Risk ratio (95% CI)** |
| --- | --- | --- | --- | --- | --- | --- |
| **≥5%** | Garvey 2025 | CagriSema vs semaglutide 2.4 mg | 2057/2108 (97.6) | 271/302 (89.7) | Reconstructed | 1.09 (1.05 to 1.13) |
|  | Yamauchi 2026 | CagriSema vs semaglutide 2.4 mg | 156/164 (95.1) | 132/167 (79.0) | Reconstructed | 1.20 (1.10 to 1.31) |
|  | Davies 2025 | CagriSema vs placebo | 811/904 (89.7) | 91/302 (30.1) | Reconstructed | 2.98 (2.50 to 3.54) |
|  | Garvey 2025 | CagriSema vs placebo | 2057/2108 (97.6) | 194/705 (27.5) | Reconstructed | 3.55 (3.15 to 4.00) |
| **≥10%** | Frias 2023 | CagriSema vs semaglutide 2.4 mg | 20/28 (71.4) | 4/29 (13.8) | Observed | 5.18 (2.02 to 13.25) |
|  | Garvey 2025 | CagriSema vs semaglutide 2.4 mg | 1914/2108 (90.8) | 227/302 (75.2) | Reconstructed | 1.21 (1.13 to 1.29) |
|  | Yamauchi 2026 | CagriSema vs semaglutide 2.4 mg | 134/164 (81.7) | 82/167 (49.1) | Reconstructed | 1.66 (1.40 to 1.97) |
|  | Davies 2025 | CagriSema vs placebo | 670/904 (74.1) | 27/302 (8.9) | Reconstructed | 8.29 (5.77 to 11.91) |
|  | Garvey 2025 | CagriSema vs placebo | 1914/2108 (90.8) | 82/705 (11.6) | Reconstructed | 7.81 (6.37 to 9.57) |
| **≥15%** | Frias 2023 | CagriSema vs semaglutide 2.4 mg | 15/28 (53.6) | 0/29 (0.0) | Observed | 32.07 (2.01 to 511.56) |
|  | Garvey 2025 | CagriSema vs semaglutide 2.4 mg | 1651/2108 (78.3) | 154/302 (51.0) | Reconstructed | 1.54 (1.37 to 1.72) |
|  | Yamauchi 2026 | CagriSema vs semaglutide 2.4 mg | 98/164 (59.8) | 50/167 (29.9) | Reconstructed | 2.00 (1.53 to 2.60) |
|  | Davies 2025 | CagriSema vs placebo | 466/904 (51.5) | 5/302 (1.7) | Reconstructed | 31.14 (13.02 to 74.43) |
|  | Garvey 2025 | CagriSema vs placebo | 1651/2108 (78.3) | 33/705 (4.7) | Reconstructed | 16.73 (11.98 to 23.36) |
| **≥20%** | Garvey 2025 | CagriSema vs semaglutide 2.4 mg | 1269/2108 (60.2) | 86/302 (28.5) | Reconstructed | 2.11 (1.76 to 2.54) |
|  | Yamauchi 2026 | CagriSema vs semaglutide 2.4 mg | 64/164 (39.0) | 32/167 (19.2) | Reconstructed | 2.04 (1.41 to 2.94) |
|  | Davies 2025 | CagriSema vs placebo | 264/904 (29.2) | 1/302 (0.3) | Reconstructed | 88.19 (12.43 to 625.74) |
|  | Garvey 2025 | CagriSema vs placebo | 1269/2108 (60.2) | 12/705 (1.7) | Reconstructed | 35.37 (20.16 to 62.04) |

**Supplementary Table S7.** Event counts underlying every safety estimate. For each trial and each arm, the number of participants with at least one event and the safety analysis-set denominator, with the trial-level risk ratio. Outcomes with no events in either arm are shown as not estimable and did not contribute to the corresponding pooled estimate.

| **Outcome** | **Trial** | **Comparison** | **CagriSema n/N (%)** | **Comparator n/N (%)** | **Risk ratio (95% CI)** |
| --- | --- | --- | --- | --- | --- |
| **Any adverse event** | Enebo 2021 | CagriSema vs semaglutide 2.4 mg | 12/12 (100.0) | 23/24 (95.8) | 1.04 (0.96 to 1.13) |
|  | Frias 2023 | CagriSema vs semaglutide 2.4 mg | 21/31 (67.7) | 22/31 (71.0) | 0.95 (0.69 to 1.33) |
|  | Garvey 2025 | CagriSema vs semaglutide 2.4 mg | 1943/2106 (92.3) | 271/302 (89.7) | 1.03 (0.99 to 1.07) |
|  | Yamauchi 2026 | CagriSema vs semaglutide 2.4 mg | 143/164 (87.2) | 141/167 (84.4) | 1.03 (0.95 to 1.13) |
|  | Davies 2025 | CagriSema vs placebo | 815/904 (90.2) | 258/302 (85.4) | 1.06 (1.00 to 1.11) |
|  | Garvey 2025 | CagriSema vs placebo | 1943/2106 (92.3) | 580/705 (82.3) | 1.12 (1.08 to 1.16) |
| **Serious adverse events** | Frias 2023 | CagriSema vs semaglutide 2.4 mg | 0/31 (0.0) | 2/31 (6.5) | 0.20 (0.01 to 4.00) |
|  | Garvey 2025 | CagriSema vs semaglutide 2.4 mg | 206/2106 (9.8) | 15/302 (5.0) | 1.97 (1.18 to 3.28) |
|  | Yamauchi 2026 | CagriSema vs semaglutide 2.4 mg | 6/164 (3.7) | 11/167 (6.6) | 0.56 (0.21 to 1.47) |
|  | Davies 2025 | CagriSema vs placebo | 94/904 (10.4) | 39/302 (12.9) | 0.81 (0.57 to 1.14) |
|  | Garvey 2025 | CagriSema vs placebo | 206/2106 (9.8) | 43/705 (6.1) | 1.60 (1.17 to 2.20) |
| **Gastrointestinal adverse events** | Enebo 2021 | CagriSema vs semaglutide 2.4 mg | 11/12 (91.7) | 19/24 (79.2) | 1.16 (0.89 to 1.51) |
|  | Frias 2023 | CagriSema vs semaglutide 2.4 mg | 18/31 (58.1) | 10/31 (32.3) | 1.80 (1.00 to 3.25) |
|  | Garvey 2025 | CagriSema vs semaglutide 2.4 mg | 1676/2106 (79.6) | 223/302 (73.8) | 1.08 (1.00 to 1.16) |
|  | Yamauchi 2026 | CagriSema vs semaglutide 2.4 mg | 87/164 (53.0) | 85/167 (50.9) | 1.04 (0.85 to 1.28) |
|  | Davies 2025 | CagriSema vs placebo | 655/904 (72.5) | 104/302 (34.4) | 2.10 (1.79 to 2.47) |
|  | Garvey 2025 | CagriSema vs placebo | 1676/2106 (79.6) | 281/705 (39.9) | 2.00 (1.82 to 2.19) |
| **Discontinuation due to adverse events** | Frias 2023 | CagriSema vs semaglutide 2.4 mg | 0/31 (0.0) | 1/31 (3.2) | 0.33 (0.01 to 7.88) |
|  | Garvey 2025 | CagriSema vs semaglutide 2.4 mg | 125/2106 (5.9) | 11/302 (3.6) | 1.63 (0.89 to 2.98) |
|  | Yamauchi 2026 | CagriSema vs semaglutide 2.4 mg | 3/164 (1.8) | 4/167 (2.4) | 0.76 (0.17 to 3.36) |
|  | Davies 2025 | CagriSema vs placebo | 76/904 (8.4) | 9/302 (3.0) | 2.82 (1.43 to 5.56) |
|  | Garvey 2025 | CagriSema vs placebo | 125/2106 (5.9) | 25/705 (3.5) | 1.67 (1.10 to 2.55) |
| **Injection-site reactions** | Enebo 2021 | CagriSema vs semaglutide 2.4 mg | 6/12 (50.0) | 7/24 (29.2) | 1.71 (0.74 to 3.98) |
|  | Frias 2023 | CagriSema vs semaglutide 2.4 mg | 1/31 (3.2) | 0/31 (0.0) | 3.00 (0.13 to 70.92) |
|  | Garvey 2025 | CagriSema vs semaglutide 2.4 mg | 256/2106 (12.2) | 8/302 (2.6) | 4.59 (2.29 to 9.18) |
|  | Yamauchi 2026 | CagriSema vs semaglutide 2.4 mg | 15/164 (9.1) | 4/167 (2.4) | 3.82 (1.29 to 11.26) |
|  | Davies 2025 | CagriSema vs placebo | 44/904 (4.9) | 0/302 (0.0) | 29.80 (1.84 to 482.41) |
|  | Garvey 2025 | CagriSema vs placebo | 256/2106 (12.2) | 21/705 (3.0) | 4.08 (2.64 to 6.32) |
| **Neoplasms** | Garvey 2025 | CagriSema vs semaglutide 2.4 mg | 134/2106 (6.4) | 20/302 (6.6) | 0.96 (0.61 to 1.51) |
|  | Yamauchi 2026 | CagriSema vs semaglutide 2.4 mg | 11/164 (6.7) | 9/167 (5.4) | 1.24 (0.53 to 2.92) |
|  | Davies 2025 | CagriSema vs placebo | 63/904 (7.0) | 20/302 (6.6) | 1.05 (0.65 to 1.71) |
|  | Garvey 2025 | CagriSema vs placebo | 134/2106 (6.4) | 31/705 (4.4) | 1.45 (0.99 to 2.12) |
| **Serious adverse events** | Enebo 2021 | CagriSema vs semaglutide 2.4 mg | 0/12 (0.0) | 0/24 (0.0) | Not estimable |
| **Discontinuation due to adverse events** | Enebo 2021 | CagriSema vs semaglutide 2.4 mg | 0/12 (0.0) | 0/24 (0. |  |

**Supplementary Figure S1.** Forest plots of safety outcomes for CagriSema versus semaglutide 2.4 mg and versus placebo. (a) Any adverse event. (b) Serious adverse events. (c) Gastrointestinal adverse events. (d) Discontinuation due to adverse events. (e) Injection-site reactions. (f) Neoplasms. Risk ratios are plotted on a logarithmic scale; a risk ratio below 1 favours CagriSema. Event counts and denominators for every trial are given in Supplementary Table S7. Plot conventions as in Figure 3.

Footnote: Columns to the left of each plot give the estimated mean change from baseline in each arm with its reported measure of variability, labelled SD or SE, and the analysis denominator; NR denotes that the trial reported no arm-level measure of variability, in which case the standard error used in pooling was derived from the published confidence interval around the treatment difference.

Columns to the left of each plot give the number of participants with the event and the analysis denominator in each arm. Counts are observed for Frias 2023 and reconstructed from model-based estimated proportions for the remaining trials, as detailed in Supplementary Table S6.

**Supplementary Figure S2.** Forest plots of patient-reported physical function for CagriSema versus semaglutide 2.4 mg and versus placebo. (a) Change in IWQOL-Lite-CT Physical Function score. (b) Change in SF-36v2 Physical Functioning score. Garvey 2025 reports both instruments only against placebo and therefore contributes to the placebo stratum only. Arm-level inputs are given in Supplementary Table S5. Plot conventions as in Figure 3.

Footnote: Columns to the left of each plot give the estimated mean change from baseline in each arm with its reported measure of variability, labelled SD or SE, and the analysis denominator; NR denotes that the trial reported no arm-level measure of variability, in which case the standard error used in pooling was derived from the published confidence interval around the treatment difference.

**Supplementary Figure S3.** Leave-one-out sensitivity analyses for efficacy outcomes, CagriSema versus semaglutide 2.4 mg, for every outcome informed by three or more trials. (a) Percentage change in body weight. (b) Absolute change in body weight. (c) Change in body-mass index. (d) Change in glycated haemoglobin. (e) Change in fasting plasma glucose. (f) At least 10% body-weight reduction. (g) At least 15% body-weight reduction. Each row shows the pooled estimate with the named trial omitted; the diamond and shaded band show the estimate and confidence interval with all trials included.

**Supplementary Figure S4.** Leave-one-out sensitivity analyses for safety outcomes, CagriSema versus semaglutide 2.4 mg. (a) Any adverse event. (b) Serious adverse events. (c) Gastrointestinal adverse events. (d) Discontinuation due to adverse events. (e) Injection-site reactions. Plot conventions as in Supplementary Figure S3.
